# Quantitative trait loci associated with drought stress tolerance in wheat primed with zinc oxide nanoparticles at seed germination and seedling stages

**DOI:** 10.1038/s41598-026-43113-7

**Published:** 2026-04-06

**Authors:** Mennatalla R. I. Mahmoud, Ahmed Sallam, Mohamed A. Karam, Yasser S. Moursi

**Affiliations:** 1 Department of Botany, Faculty of Science, Fayoum University, Fayoum, 63514 Egypt; 2 School of Biotechnology, Badr University in Assiut (BUA), Assiut, Egypt; 3 Department of Genetics, Faculty of Agriculture, Assiut University, Assiut, 71526 Egypt

**Keywords:** Germination and seedling traits, Drought-responsive genes, Quantitative trait loci (QTLs), Wheat (triticum aestivum L.), Zinc oxide nanoparticles, Nano-priming, Biotechnology, Genetics, Molecular biology, Plant sciences

## Abstract

Wheat is a major global staple crop, yet its productivity and nutritional quality are increasingly threatened by drought, raising serious food security concerns. Nanotechnology, particularly zinc oxide (ZnO) nanoparticle-based nano-priming, offers a promising strategy to improve germination, growth, and stress tolerance. Sixty-seven genotypes (65 double haloid (DH) lines + two parental lines) were phenotypically evaluated under four treatments: control (C), drought (D; PEG = 18 %), ZnO-NPs priming, and combined drought plus ZnO-NPs (D + ZnO-NPs). Twenty-two morphological traits were assessed, and QTL mapping was conducted using 3,567 SNP markers. Genotypes exhibited substantial variation, and treatments differed significantly across traits. Drought stress severely reduced performance, whereas ZnO-NPs priming improved germination and seedling establishment under both NP and NP + D conditions. The multi‑trait selection index was applied to all 65 lines, from which the ten most drought‑tolerant genotypes were identified. Of the 65 lines, 63 DH lines were used for QTL detection. Single-marker analysis revealed 12 QTLs for 22 traits across four environments. Functional annotation of 30 candidate genes indicated roles in transcriptional regulation, signal transduction, metabolism, RNA processing, and stress adaptation. Integrating molecular tools with nanotechnology provides a robust framework for wheat improvement. The identified superior genotypes, carrying key alleles for drought tolerance, represent valuable resources for breeding drought-resilient cultivars.ِ

**Supplementary Information:**

The online version contains supplementary material available at 10.1038/s41598-026-43113-7.

## Introduction

Climate change poses significant challenges to global ecosystems, water resources, and agriculture^1^. Wheat (Triticum aestivum L., 2n = 6x = 42, AABBDD) is one of the most widely cultivated staple cereal crops worldwide, playing a pivotal role in global food security and the economy^2^. It nourishes approximately 2.5 billion people and supplies key macronutrients and micronutrients^3–5^. In Egypt, wheat underpins national food systems and livestock feed through straw; it accounts for approximately 10 % of total agricultural output and approximately 20 % of agricultural imports, highlighting its strategic importance for national food security^6^. However, its productivity is increasingly threatened by frequent and severe droughts intensified by climate change^7–9^. The growing frequency of drought has caused major ecological and economic impacts, with substantial agricultural losses in recent decades and freshwater availability projected to decline by up to 50 % by 2050^10,11^. The extent of drought damage depends on its intensity, duration, crop growth stage, and the genetic makeup of the cultivar^12–14^. By 2050, the global population is projected to reach around 9.9 billion, intensifying the demand for food and nutrition^15,16^. Consequently, global wheat demand is expected to rise substantially, as overall food production will need to increase by around 60 % by 2050 to feed the growing population, at a time when water supplies are projected to decline in many regions, threatening agricultural productivity and food security^17^.

Given these challenges, evaluating wheat genetic resources for future improvement is crucial^18,19^. There is considerable potential to enhance wheat productivity by integrating pre-breeding materials and existing cultivars into genomics-assisted breeding programs^20^. Developing drought-tolerant wheat varieties is increasingly important under the growing threat of climate change and preserving as well as exploiting the wide genetic diversity present in wheat germplasm is essential to ensure sustained production and global food security. This diversity provides a broad foundation for drought adaptation through variation in root architecture, water-use efficiency, and drought-responsive metabolic pathways, thereby facilitating the selection and breeding of cultivars with enhanced drought tolerance and stable performance under water-limited conditions^21–23^. Harnessing this variability allows breeders to develop resilient wheat varieties capable of maintaining yield and quality amid increasing climatic variability^24^.

Nanotechnology offers promising applications in this regard, using nanoparticles (1–100 nm) with unique physicochemical properties to enhance plant growth, yield, and stress tolerance^25–27^. Among various application methods, seed nano-priming is still in its early stages but has shown great potential in improving germination and plant performance under adverse conditions^28–31^. Seed priming alleviates drought stress by enhancing the mobilization of seed reserves and activating metabolic processes before germination, leading to improved and more uniform seedling establishment^26,27,32,33^. Nano-priming, though still underexplored in wheat, has demonstrated promising effects on germination, seedling vigor, and stress tolerance. Among micronutrients, zinc (Zn) is vital for both human and plant health, acting as a cofactor in numerous enzymatic and regulatory functions and ranking as the fourth yield-limiting nutrient after N, P, and K^34–39^. Zinc oxide nanoparticles (ZnO-NPs) have gained attention for their unique physicochemical properties and their ability to enhance seedling vigor, growth, and stress tolerance^28,40–42^. Furthermore, ZnO nano-priming can modulate stress- and defense-related gene expression, thereby improving stress tolerance and potentially reducing reliance on chemical pesticides^43,44^.

Advanced wheat breeding lines exposed to drought stress have shown substantial yield reductions compared with control conditions, emphasizing the urgent need to develop drought-resilient varieties to sustain global food production^2^. However, the complex genetic basis of drought tolerance poses a major challenge in breeding drought-resistant cultivars. Recent advances in molecular breeding techniques, including genomic selection (GS), genome-wide association studies (GWAS), and quantitative trait loci (QTL) mapping, have provided powerful tools for improving complex quantitative traits^13^. The development of high-density linkage maps in wheat has enabled the dissection of the genetic architecture underlying drought adaptation, and numerous QTLs associated with traits such as coleoptile length, root architecture, plant biomass, membrane stability, water-soluble carbohydrates, and grain yield have been identified under drought stress^45–50^. These findings have significantly improved our understanding of the genetic control of germination and early seedling development under water deficit, and modern gene-editing approaches such as CRISPR–Cas9 hold promise for further enhancing drought tolerance in wheat^48,49^.

Given the increasing climatic variability, developing drought-tolerant wheat hybrids with superior germination and seedling establishment has become a key breeding priority. However, evaluating multiple, often correlated traits can lead to multicollinearity issues, complicating selection decisions. Multi-trait selection approaches, such as the Multi-trait Genotype–Ideotype Distance Index (MGIDI), can overcome these challenges by integrating trait information through factor analysis and ranking genotypes based on overall performance^51,52^.

Although numerous studies have explored the ameliorative effects of ZnO nanoparticles on wheat performance under drought stress, most have evaluated a narrow range of concentrations, priming durations, or developmental stages. Moreover, the responses of different wheat genotypes during germination and early seedling growth remain poorly understood. Therefore, this study aimed to (i) investigate the effects of drought stress and ZnO‑NPs seed priming on seed germination and early seedling development in a wheat double‑haploid (DH) population, (ii) map the genomic regions (QTLs) and candidate genes associated with germination and seedling traits under both control and drought conditions, and (iii) identify the most tolerant and sensitive genotype.

## Materials and methods

### Plant material

The plant material used in this study consisted of 65 double-haploid lines of wheat derived from two parents, TRI-11082 (GDR variety Hatri) x TRI-5645 (an Iranian landrace), the parental lines were selected from a diverse set originating from different geographical origins^53^. The double haploid (DH) population was generated from the corresponding F₁ hybrids through anther culture, and the DH lines were subsequently propagated under greenhouse conditions by the company Saaten Union Biotec GmbH, Leopoldshöhe, Germany, and propagated in the greenhouse^53^. The parental lines exhibited contrasting responses to drought stress, with TRI‑11802 identified as (drought‑tolerant), whereas TRI‑5645 was characterized as (drought‑sensitive). The seeds of the accessions used in the current study, were obtained from the gene bank at Leibniz Institute of Plant Genetics and Crop Plant Research (IPK), Gatersleben, Germany.

## Experimental layout

### Preliminary experiment for identification of the optimum PEG-6000 concentration

To determine an appropriate concentration of polyethylene glycol-6000 (PEG-6000) for imposing drought stress and enabling discrimination between control and stress treatments, as well as among genotypes, a series of PEG-6000 solutions (10 %, 15 %, 18 %, and 20 % w/v) was prepared. Five randomly selected genotypes were initially evaluated across these concentrations. Based on a preliminary screening using seedling growth traits, the tested PEG-6000 concentrations induced distinct levels of growth inhibition. Among these concentrations, 18 % PEG-6000 provided a stress intensity that allowed clear differentiation between control and drought-treated seedlings while maintaining sufficient seedling viability for comparative evaluation among genotypes. Accordingly, the 18 % PEG-6000 concentration was selected for subsequent experiments involving the entire population.

### Preliminary experiment for identification of the optimum concentration and time of nano priming with zinc oxide nanoparticles (ZnO-NPs)

To identify the optimum concentration and time of nano priming with zinc oxide nanoparticles that show no germination of the grains and the difference between control and drought stress, as well as the variation among genotypes under the drought stress. A series of ZnO-NPs (20-30 nm) suspensions; 50 ppm, 100 ppm, and 150 ppm was prepared freshly prepared by dispersing the particles in deionized water using ultrasonic vibration (100 w, 40 kHz) for 30 minutes.

A subset of five randomly selected genotypes for priming in different concentrations of ZnO nano suspensions (50 ppm, 100 ppm, & 150 ppm) and kept on shaker at low speed during priming under various times (6 h, 12 h, and 18 h). The 12 h & 18 h priming caused germination of the grains in some genotypes and all genotypes, respectively. Whereas the 6 h was the optimum time for priming. Thus, the 6 h was applied for the whole collection. The 50 ppm and 150 ppm concentrations were initially evaluated but were not selected due to limited suitability for comparative assessment. In contrast, the 100 ppm zinc oxide nanoparticles (ZnO-NPs) concentration provided conditions that enabled distinction between control and drought-treated seedlings, as well as among genotypes under drought stress, based on seedling growth. Accordingly, the 100 ppm ZnO-NPs concentration was selected for application to the full collection.

### Pilot experiment

All genotypes were evaluated under control (0 % PEG-6000) and drought stress (18 % PEG-6000) conditions during seed germination and seedling establishment, with and without ZnO-NPs nano priming, in a randomized complete block design (RCBD) with three replications. Twenty seeds from each genotype were washed with water and sterilized in 1 % sodium hypochlorite (NaOCl) for 10 minutes, then rinsed several times with deionized distilled water. The grains were primed by soaking for 6 h at room temperature (25 °C) in a 100 ppm ZnO-NPs solution and kept on a shaker at low speed during priming. Drought stress treatments were induced using 18 % (w/v) PEG-6000, while 0 % PEG-6000 was used as a control (unstressed grains). For each genotype, 20 grains were placed in a 9-cm petri dish on two layers of filter paper under four treatments: for unprimed conditions, T1 = control (10 ml distilled water) and T2 = drought stress (10 ml 18 % PEG-6000); for ZnO-NPs-primed conditions, T3 = control (10 ml distilled water) and T4 = drought stress (10 ml 18 % PEG-6000). The petri dishes were incubated in a growth chamber at 20 °C in darkness.

### Phenotypic traits scoring

Seed germination was scored at 24 h intervals daily for up to 12 days. Seeds were considered germinated when the radicle reached at least 2 mm in length. After 12 days, the experiment was terminated. Several germination and seedling establishment parameters were scored according to International Seed Testing Association (ISTA) rules.

Twenty-two morphological germination and seedling establishment parameters were scored from three biological replicates for each accession under each treatment. The names and abbreviations of each trait are listed in Table 1. Seed germination parameters such as final and initial germination percentage (FG % and IG %), germination pace (GP), mean germination time (MGT), mean germination rate (MGR), uncertainty of the germination process (U), synchrony of germination (Z), coefficient of variation of germination time (CVt), and seed vigor index (SVI) were calculated for the four treatments. Seedling establishment parameters such as shoot and root lengths (SL and RL) were manually measured (for each genotype) in centimeters (cm) in the four treatments for three plants per petri dish, from the top of the seed tip to the end of the shoot, using a scaled ruler. The shoot-root ratio (SRR) was calculated (for each genotype) as the ratio of shoot length to root length in centimeters (cm) in the four treatments. Root number (RNo) was counted (for each genotype) visually as the total number of roots on the 12^th^ day in the four treatments. Fresh biomass weight (FW) was recorded (for each genotype) in grams (g) by weighing germinated seeds (including root and shoot) in the four treatments using a sensitive digital balance (Sartorius AC 1215, Germany). Drought tolerance index (DTI) and reduction (R) were calculated (for the two treatments of unprimed and nano primed) for fresh weight (FWDTI and RFW), shoot length (SLDTI and RSL), root length (RLDTI and RRL), and root number (RNoDTI and RRNo) traits. A full description of the respective reductions, drought tolerance indices, and phenotypic trait measurements is provided in Table 1.

**Table 1:** Description of various parameters and formula equations used to study seed germination.

Abbreviation					
	Unprimed conditions		Nano primed conditions		
Treatments traits	Control	Drought	Drought and nano	Control and nano	Description
1.Final Germination Percentage (%)	FG %_C	FG %_D	FG %_DN	FG %_CN	$$FG \%=\frac{Number of germinated grains at {12}^{th}\mathrm{day} }{Total number of grains}\times 100$$
2.Initial Germination Percentage (%)	IG %_C	IG %_D	IG %_DN	IG %_CN	$$IG \%=\frac{Number of germinated grains at {1}^{th}day }{Total number of grains}\times 100$$
3.Germination Pace (%)	GP_C	GP_D	GP_DN	GP_CN	$GP=\frac{N}{\sum n\times g}\times 100$
4.Mean Germination Time (h)	MGT_C	MGT_D	MGT_DN	MGT_CN	$MGT=\sum \frac{nd}{n}$
5.Mean Germination Rate (day^-1^)	MGR_C	MGR_D	MGR_DN	MGR_CN	$MGR=\frac{1}{MGT}$
6.Uncertainity of Germination Process	U_C	U_D	U_DN	U_CN	$U=-\sum_{i=1}^{k}{f}_{i} {\mathrm{log}}_{2}{f}_{i}$, being${f}_{i}=\frac{{n}_{i}}{\sum_{i=1}^{k}{n}_{i}}$
7.Synchrony of Germination process	Z_C	Z_D	Z_DN	Z_CN	$Z=\frac{\sum_{i=1}^{k}{C}_{{n}_{i},2}}{{C}_{\sum {n}_{i},2}}$, being${C}_{ni,2}={{n}_{i}}^{({n}_{i}-1)}{/}_{2}$
8.Coefficient of Variation of Germination Time	CVt_C	CVt_D	CVt_DN	CVt_CN	$C{V}_{t}=\frac{{s}_{t}}{\overline{t} } 100$
9.Seed Vigour Index (%)	SVI_C	SVI_D	SVI_DN	SVI_CN	$SVI=Seedling Length\times G\%$
10.Fresh Weight (g)	FW_C	FW_D	FW_DN	FW_CN	The weight of germinated grains (including root and shoot).
11.Shoot Length (cm)	SL_C	SL_D	SL_DN	SL_CN	The distance between the top of the seed tip to the end of the shoot.
12.Root Length (cm)	RL_C	RL_D	RL_DN	RL_CN	The distance between the bottom of the seed tip to the end of the root.
13.Root Number	RNo_C	RNo_D	RNo_DN	RNo_CN	The actual count of the number of roots.
14.Shoot Root Ratio	SRR_C	SRR_D	SRR_DN	SRR_CN	The ratio of the shoot length to the root length.
15.Fresh Weight Drought Tolerance Index	FWDTI_D		FWDTI_DN		$FWDTI=\frac{FW under drought}{FW under control}\times 100$
16.Shoot Length Drought Tolerance Index	SLDTI_D		SLDTI_DN		$SLDTI=\frac{SL under drought}{SL under control}\times 100$
17.Root Length Drought Tolerance Index	RLDTI_D		RLDTI_DN		$RLDTI=\frac{RL under drought}{RL under control}\times 100$
18.Root Number Drought Tolerance Index	RNoDTI_D		RNoDTI_DN		$RNoDTI=\frac{RNo under drought}{RNo under control}\times 100$
19.Reduction of Fresh Weight (g)	RFW_D		RFW_DN		$RFW=FW under control- FW under drought$
20.Reduction of Shoot Length (cm)	RSL_D		RSL_DN		$RSL=SL under control- SL under drought$
21.Reduction of Root Length (cm)	RRL_D		RRL_DN		$RRL=RL under control- RL under drought$
22.Reduction of Root Number	RRNo_D		RRNo_DN		$RRNo=RRNo under control- RRNo under drought$

C = Control, D = Drought, DN = Drought and Nano, CN = Control and Nano, DTI = Drought Tolerance Index, and R = Reduction.

## Statistical analysis of the data

### Phenotypic data analysis

Analysis of variance (ANOVA) and broad-sense heritability were calculated using PLABSTAT software^54^. Two statistical models were employed. The first model analyzed morphological traits scored under the four treatments, using the following statistical model:

$${Y}_{ij}=\mu +{g}_{i}+{r}_{j}+g{r}_{ij}\left(error\right)$$

Where ${Y}_{ij}$ is the observation of genotype *i* in replication *j*, *µ* is the general average, ${g}_{i}$ and ${r}_{j}$ are the main effects of genotypes and replication, respectively. The error is the interaction between genotype *i* and replication *j*. For this data, genotypes and replications were considered random effects. Broad-sense heritability (${H}^{2}$) estimates for each trait were calculated by PLABSTAT using the following equation:

$${H}^{2}=\frac{{{\sigma }^{2}}_{G}}{{{\sigma }^{2}}_{G}+({{\sigma }^{2}}_{GR})}$$

where ${{\sigma }^{2}}_{G}$ is the genotypic variance and ${{\sigma }^{2}}_{G}+({{\sigma }^{2}}_{GR})$ is the phenotypic variance.

Second, another statistical model was used to analyze the morphological traits that were measured under control and drought stress with and without priming using the following model:

$${Y}_{ik}=\mu +{g}_{i}+{r}_{j}+{t}_{k}+t{g}_{ki}+tg{r}_{ijk}$$

where ${Y}_{ik}$ is the observation of genotype *i* in replication *j* in treatment $k$ (control vs drought) with unprimed and nano primed, $k$, $\mu$ is the general mean; ${g}_{i}$, ${r}_{j}$, and ${t}_{k}$ are the main effects of genotypes, replications, and treatments, respectively. tgik is genotype × treatment interaction. $tg{r}_{ijk}$ is genotype × replications × treatment interaction (error). Treatments were considered fixed effects, while replications and genotypes were considered random effects.

Microsoft Office Excel 365^55^ and R package, version 4.5.1^56^ were used to compute the multivariate estimates, Pearson correlation coefficient and the Principal Component Analysis (PCA) and for data visualization.

### Selection for the most tolerant and most sensitive genotypes

Phenotypic selection relied on the Multi-Trait Genotype–Ideotype Distance Index (MGIDI). To classify the lines according to MGIDI, the “metan” package^57^ in version 4.5.1 was employed. Each trait (rXij) was first standardized, followed by factor analysis to define the ideotype matrices. The MGIDI value was computed as the Euclidean distance between the genotype and the ideotype scores:

$${{\boldsymbol{M}}{\boldsymbol{G}}{\boldsymbol{I}}{\boldsymbol{D}}{\boldsymbol{I}}}_{{\boldsymbol{i}}}={\left[\sum_{{\boldsymbol{j}}=1}^{{\boldsymbol{f}}}{({{\boldsymbol{\gamma}}}_{{\boldsymbol{i}}{\boldsymbol{j}}}-{{\boldsymbol{\gamma}}}_{{\boldsymbol{j}}})}^{2}\right]}^{0.5}$$

Here, γij denotes the score of the ${i}^{th}$ genotype (*i* =1, 2...,* t*) in the ${j}^{th}$ factor *(j* =1, 2..., *f*), where *t* and *f* represent the total number of genotypes and factors, respectively. The ideal genotype is represented by ${\gamma }_{j}$ . Genotypes with lower MGIDI values are considered closer to the ideotype^57^. In this study, all quantitative morphological traits were assigned increasing values, except for seven traits that directly or indirectly influenced wheat’s response to drought stress.

The strengths and weaknesses of genotypes were evaluated by determining the share of the MGIDI value for the ${i}^{th}$ genotype that is attributed to the ${j}^{th}$ trait (${\omega }_{ij}$ ) using the following formula.

$${{\boldsymbol{\omega}}}_{{\boldsymbol{i}}{\boldsymbol{j}}}=\frac{\sqrt{{{{\boldsymbol{D}}}^{2}}_{{\boldsymbol{i}}{\boldsymbol{j}}}}}{\sum_{{\boldsymbol{j}}=1}^{{\boldsymbol{f}}}\sqrt{{{{\boldsymbol{D}}}^{2}}_{{\boldsymbol{i}}{\boldsymbol{j}}}}}$$

where, ${{D}^{2}}_{ij}$ is the distance between the ith genotype and ideal genotype for the *jth* trait. A trait with low contribution indicates that the genotypes within such trait are close to the ideal genotype. The analyses were performed in R Studio^58^ using R version 4.5.1. The metan package^57^ and ggplot2 package version 4.5.1. were used to conduct stability analysis on various models with different parameters.

### QTL mapping

A total of 3,567 SNP markers distributed across all chromosomes were used for linkage map construction, resulting in a total map length of 3150.71 cM. The A, B, and D sub-genomes held 1447 (1.44 per cM), 1755 (1.75 per cM), and 365 SNPs (0.36 per cM), respectively (Table 2). SNP markers ranged from 4 (chromosome 4D) to 373 (chromosome 1B). The genetic map was used to find significant associations between SNP markers and germination and seedling-establishment parameters.

**Table 2:** Summary of chromosome number, number of SNPs per chromosome, number of SNPs per genome, map length, and map density in a double haploid population of wheat.

Chromosome	No. of markers	Map length (cM)	Map distance (marker/cM)
-Ch1-1A	143	177.29	0.81
-Ch2-1B	373	144.39	2.58
-Ch3-1D	127	94.39	1.35
-Ch4-2A	266	281.13	0.95
-Ch5-2B	320	200.77	1.59
-Ch6-2D	85	111.91	0.76
-Ch7-3A	179	199.84	0.90
-Ch8-3B	103	174.65	0.59
-Ch9-3D	22	45.19	0.49
-Ch10-4A	88	163.54	0.54
-Ch11-4B	173	131.02	1.32
-Ch12-4D	4	22.23	0.18
-Ch13-5A	316	258.94	1.22
-Ch14-5B	299	200.54	1.49
-Ch15-5D	33	67.04	0.49
-Ch16-6A	230	173.31	1.33
-Ch17-6B	247	136.15	1.81
-Ch18-6D	67	14.54	4.61
-Ch19-7A	225	270.24	0.83
-Ch20-7B	240	184.26	1.30
-Ch21-7D	27	99.34	0.27
A genome	1447	1524.29	6.57
B genome	1755	1171.78	10.69
D genome	365	454.64	8.14
Total	3567	3150.71	25.41

Candidate QTL regions for control and drought component traits were identified using single marker analysis (SMA) with QTL ICIMapping Version 4.2.53 software. The phenotypic data means were analyzed using genetic markers, and QTL identification was performed using the Kosambi mapping function. The mapping population consisted of 63 lines. The type of population used was doubled haploids derived from F1 (F1DH). Marker positions were defined using centiMorgan (cM) units. These details clarify the population type, size, and marker information, which are critical for QTL detection and reproducibility. The optimal LOD threshold was determined using default software options (LOD = 2.5) and 1,000 permutation tests with Type I error at P < 0.05 (typically LOD ≥ 3.1–3.3). To avoid missing QTLs with minor effects, the LOD threshold for significant QTL detection was reduced to LOD = 2. All QTLs with LOD > 2 were reported.

### Candidate gene mining (gene annotation and expression)

To predict candidate genes associated with identified QTLs, flanking sequences of the closest linked markers were obtained from GrainGenes (http://graingenes.org, accessed 23-8-2025) and blasted against the Chinese Spring reference genome (IWGSC RefSeq v1.1, https://urgi.versailles.inra.fr/blast_iwgsc/blast.php, accessed 26-8-2025) and Ensembl Plants (http://plants.ensembl.org, accessed 26-8-2025). Gene annotations within the physical intervals of SNP markers were identified in Ensembl Plants (accessed 30-8-2025) and Knetminer (http://plants.Knetminer.org, accessed 2-9-2025). Gene expression values were calculated as transcripts per million (TPM) for each gene under control and abiotic stress, based on previously mapped RNA-seq data from RefSeq v1.1 (www.wheatexpression.com, accessed 5-9-2025).

## Results

### Phenotypic variation and evaluation under all treatments

All measured traits were negatively affected by drought stress under both unprimed and nano-primed conditions; however, the magnitude of reduction was generally lower in nano-primed seedlings. Substantial phenotypic variation was observed among genotypes across all treatments. The minimum, maximum, and mean values for all trait measurements across genotypes and treatments, including parental means, are summarized in Tables S1–S3.

Under control conditions, nano-priming resulted in higher mean values for some traits, specifically IG %, GP, MGR, Z, and SRR, while the remaining traits showed comparable responses between primed and unprimed treatments (Table S1). Under drought stress, nano-priming improved the mean values of most traits, except for FG %, MGT, U, and CVt, indicating an overall mitigating effect of ZnO-NPs on drought-induced reductions (Table S2). Drought tolerance indices and reduction parameters further supported the positive effect of nano-priming on all traits, with higher tolerance indices and lower reduction values observed under nano-primed conditions (Table S3).

Notably, RL values under drought conditions were higher than those under control in unprimed seedlings (Tables S1 and S2). In contrast, RNo values under drought were lower than those under control in nano-primed seedlings. These patterns indicate contrasting responses of root length and root number across treatments. However, under drought stress with nano-priming, both RL and RNo showed higher mean values compared with drought without nano-priming (Table S2).

All germination-related traits exhibited non-normal distributions under both control and drought conditions, with and without nano-priming, except for SVI (Figures S1a and S2a). In contrast, all seedling establishment traits followed normal distributions across all treatments (Figures S1b and S2b). Similarly, drought tolerance indices and reduction-related traits showed normal distributions under both unprimed and nano-primed conditions (Figure S3).

### Analysis of variance (ANOVA)

All evaluated traits showed highly significant variation across all treatments (P-value < 0.01) (Tables 3, 4, and 5). The analysis of variance (ANOVA) revealed substantial genetic differences among genotypes for all traits under all treatments (Tables 3, 4, and 5). The effect of replication was not significant, except for FG % (P-value > 0.1) and MGT (P-value > 0.05) under control unprimed conditions, FG % (P-value > 0.1) under drought primed conditions, and CVt (P-value > 0.1) under control primed conditions.

**Table 3:** Analysis of variance (Single ANOVA) & Heritability for all traits under control conditions with and without nano priming in a double haploid population of wheat.

Treatments	Control conditions							
Sources of variance	Genotypes (G)		Replicates (R)		RG		h^2^	
DF traits	66		2		132			
	Unprimed	Nano priming	Unprimed	Nano priming	Unprimed	Nano priming	Unprimed	Nano priming
FW	199.05**	233.28**	1.1	1.23	0.0003	0.0002	99.5	99.57
SL	89.91**	131.49**	0.64	1.55	0.0214	0.017	98.89	99.24
RL	204.93**	185.20**	1.28	0.39	0.0258	0.0247	99.51	99.46
RNo	24.31**	16.43**	0.1	0.55	0.0098	0.0074	95.89	93.91
FG %	16.03**	60.56**	2.34+	0.52	1.6834	1.4233	93.76	98.35
IG %	26.74**	316.75**	0.05	0.25	4.5005	0.5378	96.26	99.68
GP	31.16**	220.72**	0.15	0.52	1.3518	0.2575	96.79	99.55
SVI	67.84**	70.70**	2.06	0.64	2014.787	2167.5319	98.53	98.59
SRR	145.22**	101.72**	0.48	0.36	0.0006	0.0008	99.31	99.02
MGR	23.95**	67.49**	1.22	0.66	0.0002	0.0001	95.82	98.52
MGT	99.13**	199.04**	4.34*	0.53	0.0674	0.0528	98.99	99.5
U	15.89**	562.66**	1.38	0.44	0.0032	0.0001	93.71	99.82
Z	12.67**	225.48**	1.03	1.89	0.0009	0.0001	92.11	99.56
CVt	12.79**	2127.80**	0.88	2.54+	7.4495	0.0386	92.18	99.95

FW = Fresh Weight, SL = Shoot Length, RL = Root Length, RNo = Root Number, FG % = Final Germination Percentage, IG % = Initial Germination Percentage, GP = Germination Pace, SVI = Seed Vigor Index, SRR = Shoot-Root Ratio, MGT = Mean Germination Time, MGR = Mean Germination Rate, U = Uncertainty of Germination Process, Z = Synchrony of Germination Process, and CVt = Coefficient of Variation of Germination Time. The degree of significance is indicated as *p,0.05; **p,0.01; ***p,0.001.

**Table 4:** Analysis of variance (Single ANOVA) & Heritability for all traits under drought conditions with and without nano priming in a double haploid population of wheat.

Treatments	Drought conditions							
Sources of variance	Genotypes (G)		Replicates (R)		RG		h^2^	
DF traits	66		2		132			
	Unprimed	Nano priming	Unprimed	Nano priming	Unprimed	Nano priming	Unprimed	Nano priming
FW	108.79**	128.29**	0.28	1.23	0.0002	0.0002	99.08	99.22
SL	38.30**	125.37**	0.82	0.2	0.0253	0.012	97.39	99.2
RL	141.03**	117.31**	1.13	0.56	0.0142	0.0201	99.29	99.15
RNo	9.16**	30.26**	0.48	0.53	0.0172	0.0065	89.08	96.69
FGP	42.61**	39.58**	0.36	2.83+	1.5034	2.2926	97.65	97.47
IGP	21.42**	37.34**	0.23	0.45	10.1449	6.0977	95.33	97.32
GP	12.21**	28.48**	0.73	1.34	7.008	2.9699	91.81	96.49
SVI	86.63**	60.15**	0.25	0.79	804.1835	1744.9113	98.85	98.34
SRR	35.52**	56.45**	1.42	0.08	0.0002	0.0002	97.18	98.23
MGR	12.21**	70.34**	0.73	0.46	0.0007	0.0001	91.81	98.58
MGT	21.62**	39.93**	0.56	0.6	0.9498	0.454	95.37	97.5
U	11.18**	30.89**	0.51	0.55	0.0074	0.0025	91.05	96.76
Z	11.76**	20.75**	0.34	1.46	0.0017	0.001	91.49	95.18
CVt	6.27**	19.74**	0.61	0.11	16.5752	6.4362	84.05	94.93

FW = Fresh Weight, SL = Shoot Length, RL = Root Length, RNo = Root Number, FG % = Final Germination Percentage, IG % = Initial Germination Percentage, GP = Germination Pace, SVI = Seed Vigor Index, SRR = Shoot-Root Ratio, MGT = Mean Germination Time, MGR = Mean Germination Rate, U = Uncertainty of Germination Process, Z = Synchrony of Germination Process, and CVt = Coefficient of Variation of Germination Time. The degree of significance is indicated as *p,0.05; **p,0.01; ***p,0.001.

**Table 5:** Analysis of variance (Single ANOVA) & Heritability for drought tolerance index and reduction related traits under unprimed and nano priming conditions in a double haploid population of wheat.

Sources of variance	Genotypes (G)		Replicates (R)		RG		h^2^	
DF	66		2		132			
Treatments traits	Unprimed	Nano priming	Unprimed	Nano priming	Unprimed	Nano priming	Unprimed	Nano priming
FWDTI	68.31**	114.88**	0.01	0.35	1.9984	1.7128	98.54	99.13
SLDTI	50.30**	76.16**	0.28	0.25	1.3470	1.1461	98.01	98.69
RLDTI	268.54**	129.81**	0.55	0.55	2.9667	8.1071	99.63	99.23
RNoDTI	6.21**	28.26**	0.44	0.23	11.2689	4.9902	83.88	96.46
RFW	77.67**	91.57**	0.15	0.30	0.0005	0.0005	98.71	98.91
RSL	39.07**	68.52**	0.49	0.40	0.0599	0.0368	97.44	98.54
RRL	133.98**	119.37**	2.90+	1.00	0.0397	0.0418	99.25	99.16
RRNo	4.67**	25.22**	0.16	0.06	0.0337	0.0107	78.56	96.03

FWDTI = Fresh Weight Drought Tolerance Index, SLDTI = Shoot Length Drought Tolerance Index, RLDTI = Root Length Drought Tolerance Index, RNoDTI = Root Number Drought Tolerance Index, RFW = Reduction of Fresh Weight, RSL = Reduction of Shoot Length, RRL = Reduction of Root Length, and RRNo = Reduction of Root Number.

With and without nano-priming, heritability estimates for traits under control conditions were higher than their corresponding values under drought (Tables 3 and 4). Under unprimed control conditions, h^2^ ranged from 92.11 for Z to 99.51 for RL, and under nano-priming control conditions, h^2^ ranged from 93.91 for RNo to 99.95 for CVt (Table 3). Similarly, under unprimed drought conditions, h^2^ ranged from 84.05 for CVt to 99.29 for RL, and under nano-priming drought conditions, h^2^ ranged from 94.93 for CVt to 99.22 for FW (Table 4). The heritability estimates for drought tolerance index and reduction traits were also high, ranging from 78.56 for RRNo to 99.63 for RLDTI under unprimed conditions, and from 96.03 for RRNo to 99.23 for RLDTI under nano-primed conditions (Table 5).

### Correlation analyses

Pearson’s correlation analysis revealed clear and consistent relationships among traits across all treatments, reflecting differential responses to drought stress with and without nano-priming. Under control conditions, most germination-related traits were highly and positively correlated with each other and generally showed positive associations with seedling traits (Figures S4). In contrast, MGT consistently exhibited strong negative correlations with GP and MGR. Notably, GP and MGR showed the strongest positive association, both under control without nano-priming (GP_C and MGR_C, r = 0.97***) (Figure S4a) and under control with ZnO-NPs priming (GP_CN and MGR_CN, r = 0.99***) (Figure S4b). Under drought stress, similar correlation patterns were observed, with germination traits remaining tightly associated under both unprimed and nano-primed conditions (Figures S5). Notably, nano-priming generally strengthened positive correlations among key germination traits, such as GP and MGR, while maintaining negative associations with MGT, U, and CVt. Drought tolerance indices showed strong negative correlations with reduction-related traits and positive correlations with key seedling traits, including shoot length, under both primed and unprimed conditions (Figure S6). Overall, these results indicate that germination traits are highly interdependent, while seedling traits and drought tolerance indices exhibit consistent and biologically meaningful interrelationships across treatments.

### Principal component analysis

Principal component analysis (PCA) was summarized as correlation biplots with traits centered and scaled. Under control conditions, PC1 and PC2 explained 56.9 % and 19.2 % of the total variance, respectively, and under nano-priming, they explained 51.5 % and 17.9 %) (Figure S7). In both conditions, MGT, U, and CVt aligned on the positive side of PC1, indicating a positive association among germination timing and uniformity traits, while Z, MGR, GP, and IG % loaded on the negative side, reflecting negative associations with the first group. SVI and RL extended primarily along positive PC2, showing that seedling establishment traits contribute strongly to PC2, whereas SRR and RNo had short vectors near the origin, indicating low representation.

Under drought without priming, PC1 (33.7 %) and PC2 (17.3 %) captured most variability (Figure S8d). MGT, U, and CVt were positively associated with PC1, while Z, MGR, GP, and IG % were oriented oppositely. SVI and RL contributed along PC2, and drought-tolerance indices showed structured patterns: RSL_D and RRL_D projected toward positive PC2, whereas SLDTI_D, FWDTI_D, and RLDTI_D projected toward negative PC2. RNo_D had minimal contribution. Under drought with nano-priming, PC1 (34.1 %) and PC2 (19.4 %) retained a similar structure, with MGT, U, and CVt on the positive side and Z, MGR, GP, and IG % on the negative side of PC1 (Figure S8h). Drought-tolerance indices clustered along PC2, showing a consistent trade-off between tolerance and reduction metrics.

Overall, PCA revealed that germination-related traits drive variation along PC1, while seedling establishment and drought-tolerance indices dominate PC2, and these patterns were largely preserved under nano-priming. The observed associations among traits are supported by the PCA biplots: longer vectors indicate traits that contribute strongly to the principal components, while the angles between vectors reflect correlations; small angles indicate positive correlations, obtuse angles indicate negative correlations, and vectors near the origin indicate low representation. This justifies the stated positive and negative associations among germination and seedling traits.

### Selection for the most drought-tolerant and the most sensitive genotypes

All germination‑ and seedling‑related traits were utilized to identify both drought‑tolerant and drought‑susceptible genotypes under both drought conditions. Genotype ranking was performed using the Multi‑Trait Genotype–Ideotype Distance Index (MGIDI), which quantifies the distance between each genotype and an a priori defined ideotype. This multivariate approach integrates information from 22 traits and provides a robust, easy‑to‑interpret selection metric that avoids issues related to weighting coefficients and multicollinearity. The MGIDI index has been widely recognized as an efficient tool for identifying superior and inferior genotypes across diverse environments, allowing breeders to reliably assess drought tolerance and overall stability for use in subsequent breeding programs.

PCA retained six components (eigenvalues > 1) in both environments. Under unprimed drought, the six PCs explained 87.43 % of total variability, with contributions PC1 = 33.7 %, PC2 = 17.3 %, PC3 = 11.4 %, PC4 = 10.2 %, PC5 = 8.6 %, PC6 = 5.3 % (Figure S8c). Under nano primed drought, cumulative variance increased slightly to 83.07 %, with PC1 = 34.1 %, PC2 = 19.4 %, PC3 = 11.8 %, PC4 = 9.9 %, PC5 = 6.4 % (Figure S8g). The scree plot supported the retention of six components for MGIDI computation under unprimed drought and five components under nano‑primed drought conditions.

In unprimed drought, six coherent factors (FA1–FA6) captured the multivariate pattern: FA1 (germination dynamics: MGT, U, CVt, IG %, GP, MGR), FA2 (root traits: RRL and RLDTI), FA3 (germination/seedling traits): RL, FG %, and SVI), FA4 (shoot traits: RSL, SL, SRR, and SLDTI), FA5 (root number traits: RNo, RRNo, and RNoDTI), FA6 (fresh weight: FW, RFW, and FWDTI). Likewise, under nano priming, five major factors were detected, but with FA components showing stronger integration of vigor and biomass, particularly in FA2 and FA6: FA1 (germination: MGT, U, CVt, IG %, GP, FG %, Z, MGR), FA2 (shoot and root traits: RFW, RRL, FWDTI, and RLDTI), FA3 (root number traits: RRNo, RNo, and RNoDTI), FA4 (seedling and vigor: FW, SL, RL, and SVI), FA5 (shoot traits: RSL, SRR, and SLDTI).

**Fig 1:**
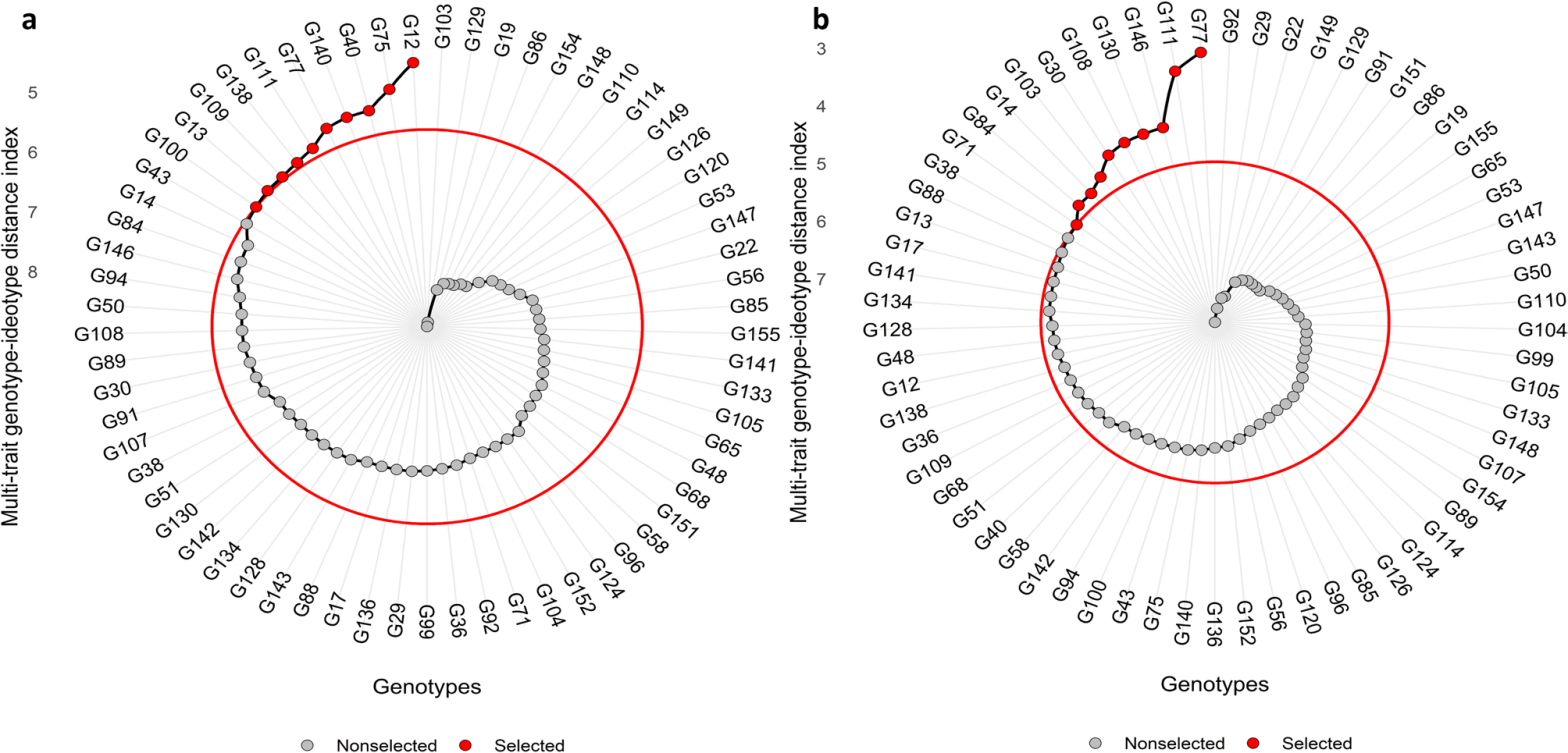
Polar plot illustrating the ranking of genotypes in ascending order based on the Multi-trait Genotype–Ideotype Distance Index (MGIDI) under drought treatment (**a**) without nano-priming and (**b**) with nano-priming. Each point represents an evaluated genotype, with selected genotypes highlighted in red in the electronic version of the article. The red circle represents the cut point established according to the applied selection pressure, separating genotypes retained for selection from thos e not selected. This visualization facilitates the identification of superior genotypes exhibiting desirable multi-trait performance under both drought-stress conditions.

Under drought stress without nano-priming, the Multi-Trait Genotype–Ideotype Distance Index (MGIDI) identified ten superior genotypes G12, G75, G40, G140, G77, G111, G138, G109, G13, and G100 as the most promising candidates for drought tolerance improvement (Fig 1a). These genotypes collectively achieved desirable selection gains (SGs) across all 22 evaluated traits, underscoring the robustness and predictive strength of MGIDI in capturing multidimensional trait performance under stress conditions. When nano-priming with zinc oxide nanoparticles was introduced, the ranking exhibited a notable shift, with the top-performing genotypes being G77, G111, G146, G130, G108, G30, G103, G14, G84, and G71 (Fig 1b).

**Fig 2:**
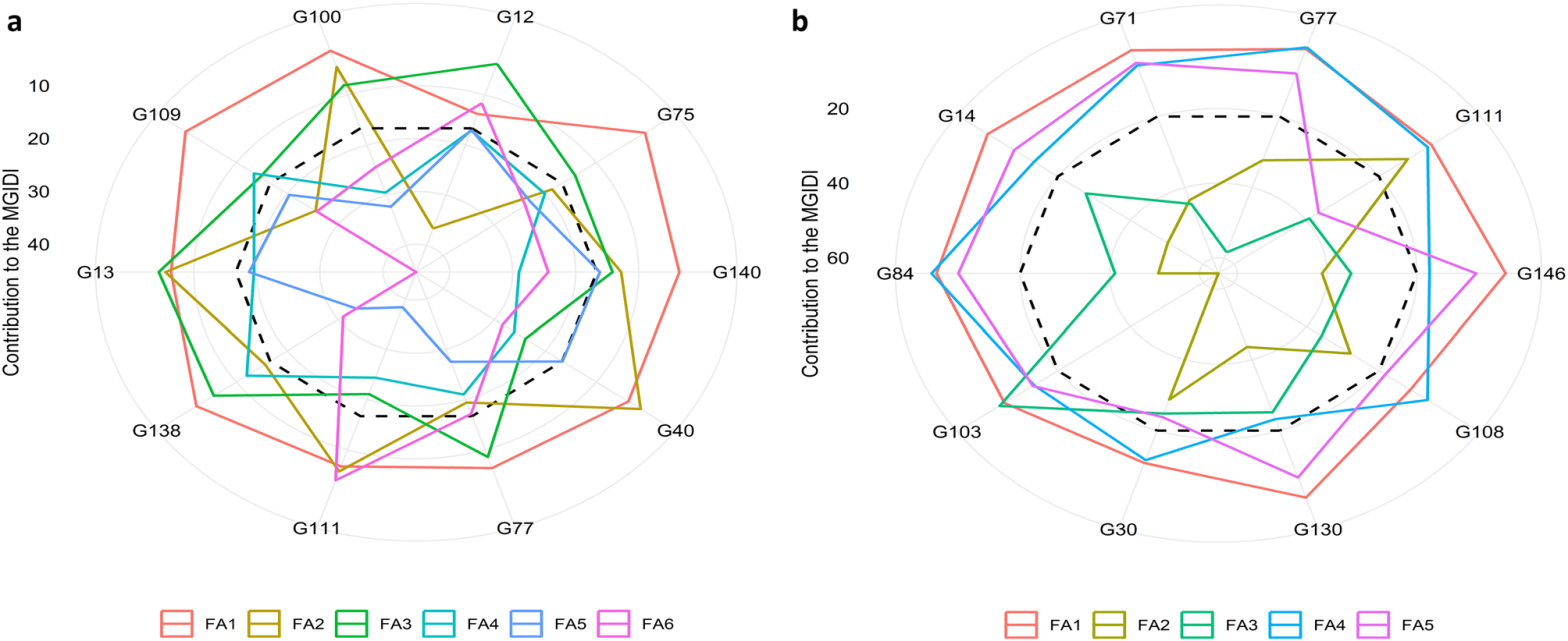
Strengths and weaknesses view of the selected genotypes showing the proportional contribution of each factor to the multi-trait genotype–ideotype distance in dex (MGIDI) under drought treatments (**a**) without nano-priming and (**b**) with nano-priming. Lower proportional contributions (positions closer to the external edge) indi cate that the traits within that factor are closer to the ideotype. The dashed line represents the theoretical contribution expected if all factors contributed equally to the M GIDI. Factor composition without nano-priming: six coherent factors (FA1–FA6) captured the multivariate pattern: FA1 (germination dynamics: MGT, U, CVt, IG %, G P, MGR), FA2 (root traits: RRL and RLDTI), FA3 (germination/seedling traits): RL, FG %, and SVI), FA4 (shoot traits: RSL, SL, SRR, and SLDTI), FA5 (root number traits: RNo, RRNo, and RNoDTI), FA6 (fresh weight: FW, RFW, and FWDTI). Factor composition with nano-priming: FA1-FA5; FA1 (germination: MGT, U, CVt, IG%, GP, FG %, Z, MGR), FA2 (shoot and root traits: RFW, RRL, FWDTI, and RLDTI), FA3 (root number traits: RRNo, RNo, and RNoDTI), FA4 (seedling and vigor: F W, SL, RL, and SVI), FA5 (shoot traits: RSL, SRR, and SLDTI).

Fig 2 shows the strengths and weaknesses view of the selected genotypes as the proportion of each factor on the computed multi-trait genotype–ideotype distance index (MGIDI) under both drought conditions. The smaller proportional contributions plotted closer to the external edge indicate that these traits already approaching the ideotype (i.e., strengths), whereas larger contributions drawn inward denote residual gaps that still separate the genotype from the ideotype (i.e., weaknesses). The dashed line marks the theoretical value if all the factors had contributed equally. FA1 (germination and seedling-related traits) are outward for most selected lines, indicating that early establishments especially of these traits are already aligned with the ideotype in top performers. This is consistent with the factor structure identified for the drought environments.

The contribution of each factor to the MGIDI index varied notably across genotypes under both drought conditions (Fig 2). The radar chart revealed clear differentiation among factors, with contributions ranked from highest (closest to the center) to lowest (farthest from the center). Under drought without nano‑priming; FA1 showed weak contributions for all selected genotypes (Fig 2a). In contrast, strong contributions to FA2 were observed in genotypes G12, G75, G77, and G109. FA3 was dominated by G40 and G111, which displayed the highest contributions. For FA4, most genotypes exhibited strong contributions except G109 and G138. Similarly, FA5 showed strong contributions for nearly all genotypes except G140, while FA6 displayed strong contributions across the selected genotypes, with the exceptions of G12 and G111. Under drought with nano‑priming, the contribution pattern shifted (Fig 2b). FA1 showed consistently weak contributions, indicating that germination traits were uniformly improved under nano‑priming and therefore contributed little to distinguishing among genotypes. FA2 exhibited strong contributions across most genotypes except G111, whereas FA3 displayed weak contributions for the majority, with G103 standing out as the primary contributor. FA4 generally showed low contributions, except for G130, which contributed strongly. In FA5, most genotypes exhibited weak contributions, except G111 and G30 representing the strong contributors. Collectively, these patterns highlight distinct shifts in factor importance under nano‑priming, reflecting enhanced coordination among traits and altered genotype performance profiles under drought stress.

**Fig 3:**
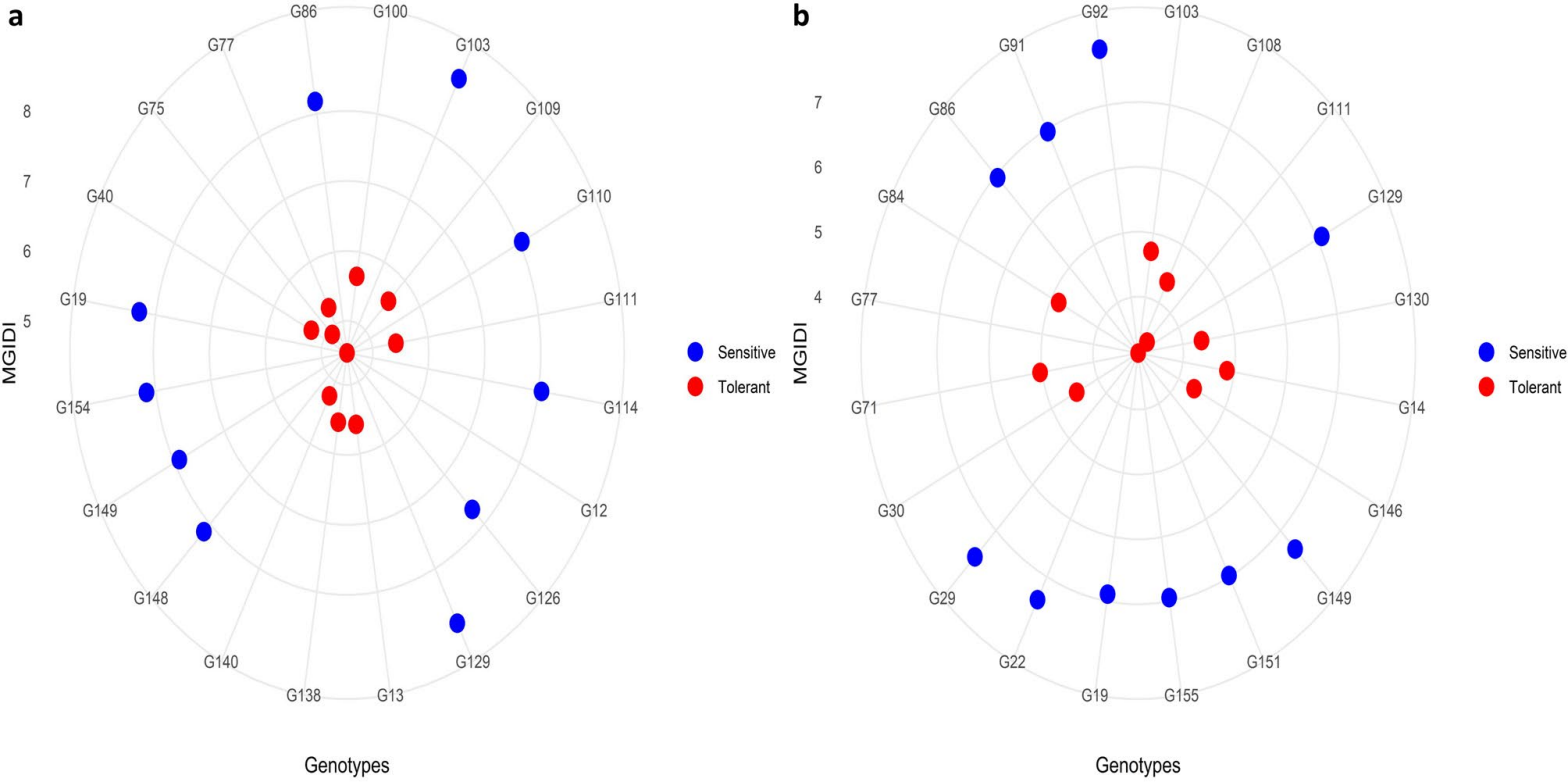
Polar plots showing the best drought-tolerant and worst drought-sensitive genotypes based on the multi-trait genotype–ideotype distance index (MGIDI) under drought treatment (**a**) without nano-priming and (**b**) with nano-priming. Genotypes with lower MGIDI values (red points) represent superior performance and greater similarity to the ideotype, whereas genotypes with higher MGIDI values (blue points) represent poor multi-trait performance under drought stress.

Comparison of MGIDI values under both drought conditions showed that nano‑priming enhanced drought tolerance in most genotypes (Table 6, Fig 3). The top‑performing genotypes (G77, G111, G13, G14, G84, G146, and G108) exhibited lower MGIDI values under nano‑priming, ranging from 3.06 (G77) to 5.06 (G13), compared with their corresponding unprimed values, which ranged from 5.26 (G77) to 6.08 (G108). This reduction indicates a clear improvement in overall performance under primed conditions. Likewise, several drought‑sensitive genotypes (e.g., G129, G19, G86, G110, G149, G53, G147, and G155) also showed improved tolerance following nano‑priming, as reflected by decreased MGIDI values that shifted from 7.18 (G155) to 8.84 (G129) under unprimed drought to 6.41 (G110) to 7.27 (G149) under nano‑priming.

**Table 6:** The most drought tolerant and sensitive genotypes based on MGIDI under drought treatment with and without nano priming in a double haploid population of wheat.

(Drought without nano priming)					(Drought with nano priming)			
No.	The most tolerant genotypes		The most sensitive genotypes		The most tolerant genotypes		The most sensitive genotypes	
	Genotype code	MGIDI	Genotype code	MGIDI	Genotype code	MGIDI	Genotype code	MGIDI
1	G12	4.49	G103	8.91	G77	3.06	G92	7.75
2	G75	4.92	G129	8.84	G111	3.34	G29	7.50
3	G140	5.20	G19	8.28	G146	4.26	G22	7.31
4	G40	5.20	G86	8.15	G130	4.29	G149	7.27
5	G77	5.26	G154	8.12	G108	4.31	G129	6.98
6	G111	5.46	G148	8.11	G30	4.38	G91	6.90
7	G138	5.53	G110	8.04	G103	4.63	G151	6.86
8	G13	5.58	G114	8.01	G14	4.76	G86	6.86
9	G109	5.57	G149	7.83	G84	4.77	G19	6.85
10	G100	5.62	G126	7.65	G71	4.96	G155	6.84
11	G43	5.66	G120	7.59	G38	4.98	G65	6.72
12	G14	5.85	G53	7.52	G88	5.01	G53	6.66
13	G84	5.86	G147	7.39	G13	5.06	G147	6.59
14	G146	5.90	G22	7.24	G17	5.07	G143	6.54
15	G94	6.00	G56	7.22	G141	5.08	G50	6.47
16	G50	6.06	G85	7.19	G134	5.09	G110	6.41
17	G108	6.08	G155	7.18	G128	5.15	G104	6.30

Overall, the analysis demonstrates that nano‑priming had a positive influence on drought tolerance across both high‑ and low‑performing genotypes, leading to improved germination efficiency and seedling establishment under stress conditions. Among the drought‑tolerant genotypes, substantial improvements in ranking were observed following nano‑priming. For example, G77, G111, G14, G84, G146, and G108, initially ranked fifth, sixth, twelfth, thirteenth, fourteenth, and seventeenth under unprimed drought conditions, improved to first, second, eighth, ninth, third, and fifth, respectively, under nano‑primed drought conditions. In contrast, several drought‑sensitive genotypes exhibited a decline in ranking following nano‑priming. G129, G19, G86, G110, G53, and G147 which ranked second, third, fourth, seventh, twelfth, and thirteenth under unprimed drought stress, were re‑ranked as fifth, ninth, eighth, sixteenth twelfth, and thirteenth, respectively, after nano‑priming. Notably, genotype G103 displayed a remarkable shift in performance. Despite being the most drought‑sensitive genotype under unprimed conditions, nano‑priming substantially improved its drought response, elevating its ranking to seventh among the most tolerant genotypes. This enhancement was reflected clearly in its MGIDI score, which decreased from 8.91 under drought without priming to 4.63 under nano‑priming, confirming a considerable improvement in its adaptive efficiency.

Collectively, nano-priming demonstrated a dual adaptive effect under drought stress: genotypes that were originally categorized as the most drought-tolerant became even more efficient in maintaining vigor and germination performance, while extremely sensitive genotypes exhibited reduced susceptibility. Notably, only one sensitive genotype underwent a substantial shift in drought response, transitioning into the group of the most tolerant following nano-priming. This shift reflects not only improved drought performance for this genotype but a true modification in drought-response status, indicating that nano-priming can readjust tolerance levels rather than merely enhance resistance.

### QTL mapping under control and drought conditions with and without priming

**Fig 4:**
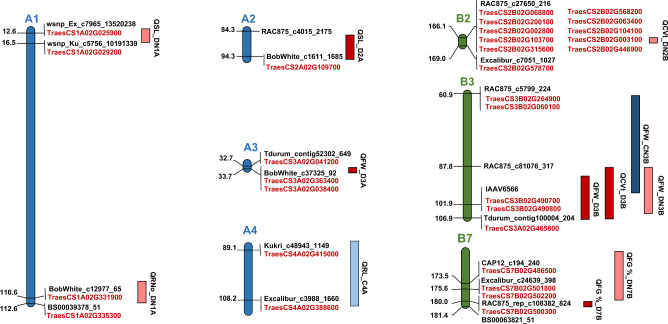
Chromosome-wise QTL mapping and localization of candidate genes associated with germination and seedling-establishment traits under control and drought conditions, with and without nano-priming. Each chromosome is represented by a vertical-colored bar (blue for A-genome chromosomes and green for B-genome chromosomes). QTLs are shown as colored blocks along the chromosomes according to the treatment under which they were detected. Candidate genes co-localized within the QTL intervals are highlighted in red. Marker names and their genetic positions (cM) are displayed to the right and left of each chromosome, respectively.

In this study, a total of 12 QTLs were found to be associated with wheat germination and seedling-establishment traits were detected, explaining phenotypic variation ranging from 3.44 % (Q CVt_DN2B) to 21.72 % (QSL_D2A). The distribution of these QTLs is shown in Fig 4. Detailed statistics, including additive effects, flanking markers, percentage of variation explained by each QTL, and LOD scores, are summarized in Table 7. LOD scores ranged from 2.06 to 14.06, with QTLs distributed across 7 chromosomes: 1A, 2A, 3A, 4A, 2B, 3B, and 7B. Chromosome 3B harbored the highest number of QTLs (4) and followed by 1A and 7B (2 QTLs). Only one additive QTL was detected on each of the following chromosomes: 2A, 3A, 4A, and 2B. Based on the proportion of phenotypic variation explained, QTLs were classified as one very strong and stable (PVE ≥ 20 %), ten majors (PVE = 10 – 20 %), and one minor (PVE ≥ 5 %).

**Table 7:** A list of QTLs for germination and seedling establishment traits in a double haploid population of wheat

	QTL	Trait name	Chromosome		Position	Left marker	Right marker	LOD	PVE (%)	Add	LeftCI	RightCI	Environment
1	QRL_C4A	RL_C	10	4A	101.00	Kukri_c48943_1149	Excalibur_c3988_1660	2.73	16.70	-1.82	89.50	113.50	Control
2	QFW_D3A	FW_D	7	3A	33.00	Tdurum_contig52302_649	BobWhite_c37325_92	2.74	14.43	-0.10	31.50	33.50	Drought
3	QFW_D3B	FW_D	8	3B	102.00	IAAV6566	Tdurum_contig100004_204	3.43	18.59	-0.12	90.50	106.50	Drought
4	QSL_D2A	SL_D	4	2A	85.00	RAC875_c4015_2175	BobWhite_c1611_1685	2.85	21.72	0.74	83.50	92.50	Drought
5	QFG %_D7B	FG %_D	20	7B	181.00	RAC875_rep_c108382_824	BS00063821_51	2.54	17.22	5.62	179.50	181.50	Drought
6	QCVt_D3B	CVt_D	8	3B	97.00	RAC875_c81076_317	IAAV6566	2.50	16.43	7.30	87.50	106.50	Drought
7	QFW_CN3B	FW_CN	8	3B	83.00	RAC875_c5799_224	RAC875_c81076_317	2.76	19.36	-0.16	62.50	98.50	Control and Nano
8	QFW_DN3B	FW_DN	8	3B	102.00	IAAV6566	Tdurum_contig100004_204	2.60	17.59	-0.11	89.50	106.50	Drought and Nano
9	QSL_DN1A	SL_DN	1	1A	13.00	wsnp_Ex_c7965_13520238	wsnp_Ku_c5756_10191339	2.51	17.00	-0.79	11.50	16.50	Drought and Nano
10	QRNo_DN1A	RNo_DN	1	1A	111.00	BobWhite_c12977_65	BS00039378_51	2.79	18.45	-0.32	102.50	110.50	Drought and Nano
11	QCVt_DN2B	CVt_DN	5	2B	168.00	RAC875_c27650_216	Excalibur_c7051_1027	14.07	3.45	17.83	166.50	168.50	Drought and Nano
12	QFG %_DN7B	FG %_DN	20	7B	175.00	CAP12_c194_240	Excalibur_c24639_398	2.07	14.19	6.08	161.50	179.50	Drought and Nano

MGT = Mean Germination Time, MGR = Mean Germination Rate, U = Uncertainty of Germination Process, CVt = Coefficient of Variation of Germination Time, FG % = Final Germination Percentage, IG % = Initial Germination Percentage, GP = Germination Pace, SVI = Seed Vigor Index, Z = Synchrony of Germination Process, FW = Fresh Weight, SL = Shoot Length, RL = Root Length, RNo = Root Number, SRR = Shoot-Root Ratio, PVE(%) = Percentage of Phenotypic Variance explained by the QTL, and Add = Additive Effect.

Under control conditions without priming, one major QTL (QRL_C4A) was identified on chromosome 4A, explaining 16.7 % of the phenotypic variation and exhibiting negative additive effects. Under control conditions with nano‑priming, another QTL (QFW_CN3B) was detected on chromosome 3B, showing a negative additive effect and accounting for 19.36 % of the variation (Table 7, Fig 4).

Under drought conditions without priming, five QTLs were identified: QFW_D3B and QCVt_D3B on chromosome 3B; QFW_D3A on chromosome 3A; QSL_D2A on chromosome 2A; and Q FG %_D7B on chromosome 7B. QFW_D3A and QFW_D3B displayed negative additive effects, explaining 14.42 % and 18.59 % of the variation, respectively, while QCVt_D3B, QSL_D2A, and QFG %_D7B showed positive additive effects, accounting for 16.42 %, 21.72 %, and 17.22 %, respectively. Under drought with nano‑priming, five QTLs were identified: QSL_DN1A and QRNo_DN1A on chromosome 1A; QFW_DN3B on chromosome 3B; QCVt_DN2B on chromosome 2B; and QFG %_DN7B on chromosome 7B. QFW_DN3B, QSL_DN1A, and Q RNo_DN1A had negative additive effects, explaining 17.58 %, 17.00 %, and 18.44 % of the variation, while QCVt_DN2B and QFG %_DN7B exhibited positive additive effects, accounting for 3.44 % and 14.19 % (Table 7, Fig 4).

### Gene annotation, expression, and mapping of the candidate genes

All flanking markers of the significant QTLs were blasted against the Ensembl Plants genomic database to identify candidate genes and their protein-coding sequences. A total of 196 distinct gene models were detected for all traits under all conditions (Table S4). These 196 candidate genes encode different proteins with diverse biological and molecular functions. Some of them function as transcription factors, ion transporters, or signaling molecules. The identified markers were anchored within 30 gene models out of these 196 genes, indicating strong consistency between the current results and the reference genomic data. Of these 45 gene models, 14 and 11 have previously been published with evidence for their association with drought tolerance and root-related traits in wheat, respectively. Chromosome 2B harbored the highest number of candidate genes. All gene models encode functional proteins except for one gene (Table S4 and S5).

Functional annotation based on BLAST analysis revealed that the drought-responsive genes identified in this study encode a diverse range of proteins associated with several biological and molecular functions. These proteins were grouped into major functional categories, including transcriptional regulation, signal transduction, metabolic and enzymatic activity, RNA processing/post-transcriptional control, hypothetical, and uncharacterized proteins. The transcription factors and regulatory proteins (five genes) play crucial roles in regulating gene expression and activating stress-responsive signaling pathways under drought conditions. The signal transduction and post-translational regulation proteins (two genes) participate in degradation and modulation of stress signaling cascades. In the metabolic and enzymatic proteins category (19 genes), enzymes such as Fatty acyl-CoA reductase, NADP-dependent oxidoreductase (two genes), Acyl-CoA dehydrogenase, Prolyl endopeptidase, GDSL esterase/lipase (two genes), O-methyltransferase, Sulfotransferase (10 genes), and Photosystem II stability/assembly factor were identified, indicating their participation in secondary metabolism, energy regulation, and Osmo protective mechanisms during drought stress. Additionally, hypothetical proteins, including DUF4220 domain-containing protein and Transmembrane protein 53, were associated with protein synthesis and mRNA processing, highlighting the importance of translational control under drought conditions. Finally, one uncharacterized protein was also detected. These may represent novel drought-responsive candidates that warrant further functional characterization.

**Fig 5:**
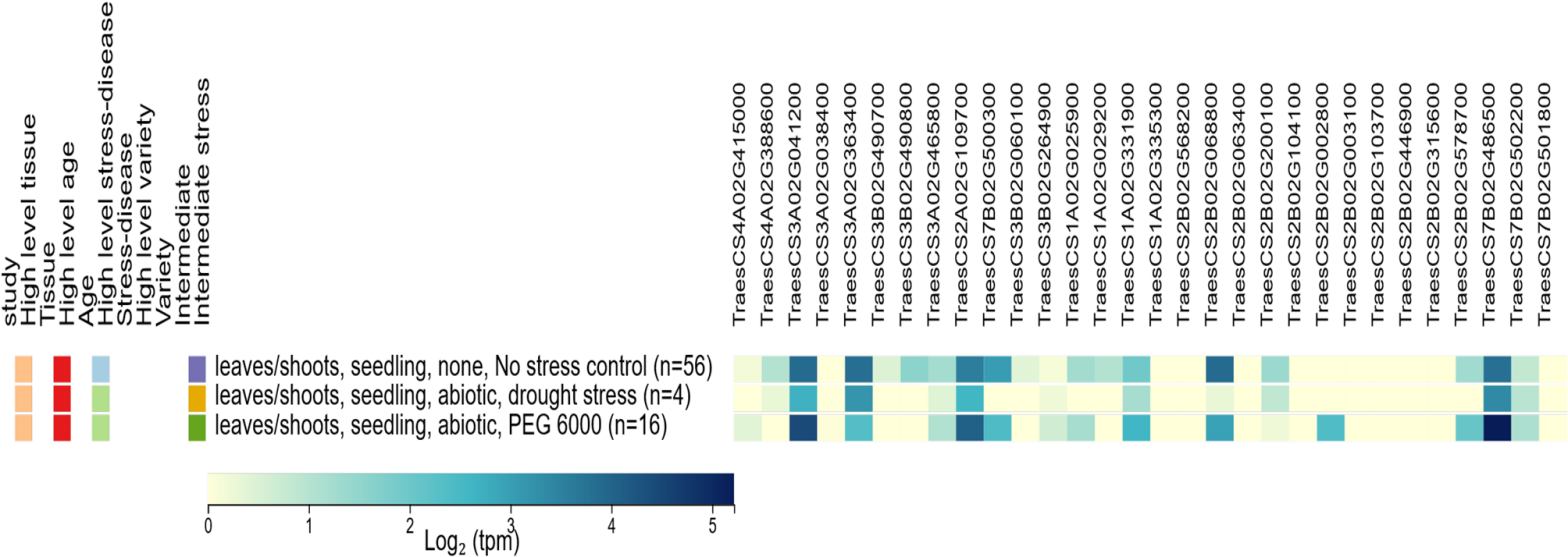
Expression profiles of the candidate genes associated with germination and seedling-establishment traits in wheat leaves/shoot tissues at the seedling stage under three conditions: control, drought, and PEG-induced osmotic stress. Expression values were retrieved from the Wheat Expression database, and normalized transcript abundance is shown for each gene across the tested stress treatments.

The expression of all gene models was obtained from the wheat expression database at the seedling stage under normal and abiotic stress conditions (Table S6, Fig 5). Among these, 10 genes were upregulated, 11 genes were downregulated, and the remaining genes were stably expressed in wheat seedlings under PEG-6000-induced drought stress, revealing three distinct categories of gene responses.

The upregulated genes could be further divided into two subgroups. The first subgroup included genes that were already expressed under normal conditions but exhibited higher transcriptional activity under drought stress, suggesting an enhanced activation of pre-existing stress-response mechanisms. For example, *TraesCS4A02G415000, TraesCS3A02G041200, TraesCS2A02G109700, TraesCS3B02G264900, TraesCS1A02G331900, TraesCS2B02G578700, TraesCS7B02G486500,* and *TraesCS7B02G502200* showed increased expression under drought, indicating their role in osmotic adjustment and antioxidant defense. The second subgroup consisted of genes that were not expressed under control conditions but were induced exclusively during drought, indicating that they are stress-responsive and specifically activated by water deficit. Examples include *TraesCS2B02G002800* which was fully induced under drought and are known to be associated with stress signaling and dehydration tolerance (Table S6).

In contrast, the downregulated genes showed two main patterns of expression change. The first pattern involved genes that were actively expressed under normal conditions but showed a marked decrease in transcription under drought stress. For instance, *TraesCS3A02G363400, TraesCS3A02G465800, TraesCS7B02G500300, TraesCS2B02G068800, and TraesCS2B02G200100,* exhibited reduced expression levels, suggesting downregulation of growth-related or metabolic pathways. The second pattern comprised genes that were expressed under control conditions but became completely silenced under drought, suggesting a full repression of their transcriptional activity, possibly as an energy-saving mechanism or as part of a regulatory shift redirecting resources toward stress tolerance pathways. Examples include *TraesCS4A02G388600, TraesCS3B02G490700, TraesCS3B02G490800, TraesCS3B02G060100,* and *TraesCS1A02G029200*, which were fully repressed under drought stress (Table S6).

Meanwhile, several genes maintained stable expression levels between control and drought conditions. Interestingly, several of these were drought-related genes that exhibited nearly constant expression despite stress exposure. This stability suggests that these genes may play a constitutive role in drought tolerance, allowing the plant to preserve essential defense and metabolic functions even under water deficit. The maintenance of steady transcriptional activity indicates a form of regulatory resilience, where the plant sustains the baseline expression of critical stress-responsive genes to ensure physiological stability and efficient adaptation to drought conditions. For example, *TraesCS1A02G335300, TraesCS2B02G568200,* *TraesCS2B02G063400*, and *TraesCS2B02G104100* showed consistent expression levels under both control and drought treatments, despite being known for their involvement in drought-response pathways. This finding suggests that their constitutive expression contributes to maintaining the plant’s defense readiness and overall resilience under stress (Table S6).

## Discussion

Seed priming is a well‑established approach for enhancing germination and early seedling growth under drought and other abiotic stresses^59^. Consistent with previous reports, ZnO‑NP nano‑priming improved germination performance and seedling establishment in both control and drought conditions^60^ (Tables S1–S3). The high heritability estimates indicate strong additive genetic control of the evaluated traits, while the consistent improvements observed under nano‑priming further support its effectiveness in enhancing seed vigor in water‑limited environments (Tables 3, 4, and5). These results align with recent findings highlighting the positive effects of ZnO‑NPs on germination, vigor, and antioxidant activity^60^, and extend previous work by jointly assessing genotype performance and drought responsiveness under nano‑priming.

Significant positive correlations were observed among germination and seedling traits across treatments, confirming that faster and more uniform germination is associated with seedling vigor. The strongest correlations were between MGR and GP under control (r = 0.97*** without ZnO‑NPs; r = 0.99*** with ZnO‑NPs priming) and drought stress (r = 1.00*** without ZnO‑NPs priming; r = 0.97*** with ZnO‑NPs priming). In contrast, MGT showed strong negative correlations with both MGR and GP (Figure S4 and S5). Correlations were generally stronger under ZnO‑NPs priming, indicating improved physiological coordination and enhanced drought tolerance. These results are consistent with previous findings that nano‑priming improves germination, seedling vigor, and antioxidant activity under drought stress^60^.

The first two principal components explained more than 50 % of the total variance across treatments, indicating that they captured the major patterns of phenotypic variation. Germination- and seedling-related traits contributed most strongly to these components, whereas root-number traits showed minimal influence. This pattern suggests that seedling establishment traits were tightly associated and dominated the first principal axis, while germination traits primarily drove variation along the second axis (Figure S7 and S8). These findings are consistent with^61^, who similarly reported that germination and seedling traits were the main contributors to phenotypic variation under PEG‑induced drought stress in wheat.

The MGIDI index proved effective for multi‑trait selection by identifying genotypes closest to the ideotype, thereby offering a balanced and objective assessment of drought tolerance (Fig 1). Under drought without nano‑priming, G12 (MGIDI = 4.49) was the most tolerant genotype, whereas G77 emerged as the most promising candidate parent, showing high drought tolerance under both unprimed and nano‑primed conditions (MGIDI = 5.26 and 3.06, respectively) (Table 6, Fig 3). As expected, tolerant genotypes exhibited lower MGIDI values than susceptible ones^62^. The most drought‑sensitive genotypes were G103 under unprimed drought (MGIDI = 8.91) and G92 under nano‑primed drought (MGIDI = 7.75), which can serve as useful checks or susceptible parents in future breeding. Because maintaining early‑stage tolerance at later developmental stages is essential, field evaluation under water‑limited conditions remains necessary. Accordingly, all genotypes are currently being tested under field drought in Egypt, following recommendations to combine controlled‑environment and field‑based selection, as suggested by Sewore and Abe^63^.

Drought tolerance is a complex polygenic trait controlled by multiple genes, making QTL analysis a valuable tool for dissecting its genetic basis^64^. This approach has been widely and successfully used to identify markers and QTLs associated with early-stage drought tolerance in major cereals such as wheat, rice, barley, and maize^65^. Single marker analysis (SMA) was performed for all 22 germination and seedling-establishment parameters under four treatments. The QTL analyses were performed using 63 genotypes, which are fewer than the population size needed for QTL identification, as it was reported that 100 - 500 individuals are required for detecting QTL^66^. Therefore, SMA in this study may have limited power for detecting minor QTLs and may produce wider confidence intervals due to the relatively small population size. Even so, the high heritability of the traits and the use of a dense marker set (~ 3,000 SNPs) provide dedicated support for the reliability of the major QTLs identified. Recent studies indicate that advanced statistical methods, such as quantile regression, can detect QTLs effectively even with small sample sizes when heritability is high, allowing robust and meaningful results despite limited genotyping^67^. Furthermore, high marker density improves mapping precision and helps offset the limitations of small population sizes^68^. Therefore, despite the reduced sample size, the combination of high heritability, dense genotyping, and appropriate analytical methods supports confidence in the validity of the detected QTLs^67^. A notable example is the QTL‑seq approach, which integrates bulked segregant analysis with whole‑genome resequencing. In this method, two bulks are formed from individuals at the phenotypic extremes, typically containing 20 – 50 plants each^67^. The original QTL‑seq study in rice successfully identified QTLs for disease resistance and seedling vigor using bulks of similar size^69^. This demonstrates that QTL mapping is feasible with fewer than 100 individuals, particularly when targeting major‑effect loci. Additionally, simulation studies and methodological research further support the effectiveness of QTL mapping in small populations. For example, mapping‑by‑selection studies in rice have shown that QTLs can be detected in populations as small as 25 plants when strong selection is applied and the trait is controlled by major loci^70^. However, these approaches are mainly effective for large‑effect QTLs, and their power to detect minor loci remains limited in small populations. Moreover, as a validation for the significant markers associated with QTL in the current study, several markers identified in this study have also been previously reported in other wheat collections under drought stress. For instance, *IAAV6566*, which was associated here with CVt and FW under both primed and unprimed drought conditions, was previously linked to drought tolerance across multiple environments in 246 spring wheat lines^71^. Likewise, *BobWhite_c1611_1685*, associated with SL in our study, was also reported in 186 spring wheat lines^72^. In addition, *BS00039378_51* showed an association with RNo under drought, consistent with findings in 299 hard winter wheat lines^73^. This validation indicates the robustness of the QTL analysis and statistical approach employed in the current study. The promising QTLs detected here are putative and require validation across diverse genetic backgrounds in large populations before their use in marker-assisted selection. Such genetic validation is essential to confirm the consistent effects of these markers^74^.

In this study, 12 QTLs associated with germination and seedling traits were identified across all treatments. Major QTLs with high PVE were mainly located on the A and B genomes. Specifically, loci were detected on chromosomes 1A (2), 2A (1), 3A (1), 4A (1), 2B (1), 3B (4), and 7B (2), linked to FW, RL, SL, RNo, FG %, and CVt (Table 7, Fig 4).

Two QTLs associated with RL and FW under control conditions, with and without nano‑priming, were mapped on chromosomes 4A and 3B. On chromosome 4A, QRL_C4A was detected at 101 cM (CI: 89.5 – 113.5 cM), close to previously reported QTLs for root depth (94 cM)^75^, grain yield (93.54 cM), and plant height (105 cM)^76^. This clustering highlights chromosome 4A as an important region regulating multiple growth‑ and yield‑related traits. On chromosome 3B, QFW_CN3B was mapped at 83 cM (CI: 62.5 – 98.5 cM) and overlapped with QTLs for plant height (75.7 cM)^77^, root depth (68.3 – 92.4 cM)^75^, and yield components (69.47 – 97.61 cM)^76^. The strong clustering of these loci within the confidence interval of QFW_CN3B indicates that chromosome 3B is a hotspot influencing early seedling vigor and key agronomic traits. The co‑localization of FW with root depth, plant height, and yield suggests possible pleiotropic effects or tightly linked loci that contribute to biomass accumulation and productivity.

Ten QTLs detected under drought, with and without nano‑priming, were mapped on chromosomes 1A, 2A, 3A, 2B, 3B, and 7B, and were associated with FW, SL, RNo, FG %, and CVt (Table 7, Fig 4). On chromosome 1A, two QTLs (QSL_DN1A and QRNo_DN1A) were detected under drought with nano‑priming, both related to seedling traits. QSL_DN1A, associated with SL, was mapped at 13.0 cM (CI: 11.5 – 16.5), while QRNo_DN1A, associated with RNo, was located at 111.0 cM (CI: 102.5 – 110.5). Notably, QSL_DN1A falls within a genomic region previously linked to several agronomic traits, including plant height (0 – 17.7 cM)^78^, kernel weight (9.6 – 12 cM)^79^, and spike‑related traits^80^. This co‑localization suggests a conserved hotspot regulating multiple growth‑ and yield‑related traits. QRNo_DN1A, mapped at 111.0 cM, lies close to QTLs previously reported for root hair length (105 – 110 cM)^81^, root depth (106.2 cM)^75^, coleoptile length (106.9 cM)^82^, thermotolerance (105.55 – 107.67 cM)^83^, and grain zinc content^84^. Such clustering indicates specific genetic control over root initiation and early seedling establishment. The overlap with grain‑zinc QTLs suggests that this region integrates responses to both drought and zinc‑related pathways, reinforcing its functional role in seedling establishment. Notably, the detection of both QSL_DN1A and QRNo_DN1A only under ZnO‑NPs priming highlights the ability of nano‑priming to stimulate shoot elongation and root development. These loci were not observed under drought alone, indicating that ZnO‑NPs priming may enhance gene expression or physiological responses that promote early seedling vigor and improve micronutrient uptake under water‑deficit conditions. On chromosome 2A, a QTL designated QSL_D2A, associated with SL, was detected under drought conditions without nano‑priming and mapped at 85.0 cM (CI: 83.5 – 92.5 cM). This region lies close to previously reported QTLs for root depth (91.7 cM)^75^, grain yield (87.8 – 88.65 cM)^85^, and spike length (83.23 cM)^86^, indicating a conserved hotspot influencing both early seedling growth and yield‑related traits in wheat. On chromosome 3A, QFW_D3A associated with FW was detected under drought without nano‑priming and mapped at 33.0 cM (CI: 31.5 – 33.5 cM). This region lies close to QTLs previously reported for days to maturity (32.2 cM)^87^ and ear emergence time (33.21 cM)^88^, suggesting a conserved genomic interval influencing early biomass accumulation and developmental timing, two key traits for drought adaptation. On chromosome 2B, QCVt_DN2B associated with CVt was detected under drought with nano‑priming and mapped at 168.0 cM (CI: 166.5 – 168.5 cM). This region lies near previously reported QTLs for plant height (15.4 – 178.2 cM)^89^, days to heading, harvest index, kernel per spike, and thousand-grain weight (162.8 – 177.7 cM)^90^, suggesting a conserved genomic hotspot influencing germination performance and key agronomic traits under drought stress. On chromosome 3B, three closely positioned QTLs related to germination and early seedling traits were detected under drought with and without nano‑priming. QFW_D3B, associated with FW, was mapped at 102.0 cM (CI: 90.5 – 106.5), while its corresponding locus under nano‑priming, QFW_DN3B, appeared within a similar interval (CI: 89.5 – 106.0). QCVt_D3B, linked to CVt, was identified at 97.0 cM (CI: 87.5 – 106.5). The proximity of these loci indicates that this region plays a significant role in regulating biomass accumulation and germination performance under drought. This interval also overlaps with several previously reported QTLs, including those for root depth (92.4 cM)^75^, grain yield (97.1 cM)^76^, plant height (94.5 – 105.5 cM)^91^, grain‑fill duration (92.9 – 118.8 cM)^87^, and grain number per spike (94.32 – 94.77 cM)^92^, confirming chromosome 3B as a significant hotspot for early vigor and key agronomic traits. These three QTLs were identified in this 3B region: two FW loci detected under primed and unprimed drought, and one germination locus detected only under unprimed drought. Their overlap indicates a stable, stress‑responsive hotspot affecting both biomass and germination, with nano‑priming modifying the expression of FW‑related loci. On chromosome 7B, two closely positioned QTLs associated with FG % were detected under both drought conditions. QFG %_D7B was mapped at 181.0 cM (CI: 179.5 – 181.5) under drought without priming, while QFG %_DN7B appeared at 175.0 cM (CI: 161.5 – 179.5) under nano‑priming. Their proximity indicates that this region plays a significant role in regulating FG % under stress, with nano‑priming potentially broadening the effective interval of the locus. This region also co‑localizes with previously reported QTLs for tiller number and thousand‑kernel weight (163.9 – 185.6 cM)^93^, suggesting a genomic hotspot influencing germination, early reproductive development, and yield‑related traits in wheat.

Candidate genes were annotated using Ensembl Plants and KnetMiner databases^94,95^, revealing that the identified genes belong to diverse functional families, including enzymatic proteins, transcription factors, and signal‑transduction components. Notably, QCVt_DN2B on chromosome 2B, detected under drought with nano‑priming, showed a strong enrichment of sulfotransferase (SOT) genes (Table S4 and S5). Eleven genes formed a functionally coherent cluster, suggesting a coordinated regulatory module influencing early seedling development. These genes clustered around marker *RAC875_c27650_216*, and most of them encode sulfotransferase (SOT) enzymes. SOTs catalyze the sulfonation of hormones and metabolites, thereby modulating their stability, transport, and biological activity, with key roles in ABA and auxin signaling, redox homeostasis, and metabolic activation during germination^96–99^. This result agrees with previous studies reporting the involvement of SOT genes in drought and heat stress tolerance in wheat^100^. Evidence from several studies linked these genes to diverse agronomic and stress-related traits, including yield components, drought tolerance, disease resistance, and reproductive development. The convergence of these associations with QTLs detected here indicates that the 2B SOT cluster may regulate germination, seedling vigor, and broader adaptive traits. Under nano-priming, enhanced germination could partly reflect SOT-mediated modulation of hormonal balance and antioxidant systems. Thus, this genomic hotspot represents a novel target for functional validation and wheat breeding. Beyond chromosome 2B, several candidate genes identified across other chromosomes encode enzymes such as fatty acyl‑CoA reductase, NADP‑dependent oxidoreductase, acyl‑CoA dehydrogenase, prolyl endopeptidase, GDSL esterase/lipase, O‑methyltransferase, and a Photosystem II stability factor. Collectively, these proteins contribute to key metabolic and redox pathways that support drought tolerance, seedling vigor, and photosynthetic stability (Table S4 and S5). The genes encoding fatty acyl‑CoA reductases and acyl‑CoA dehydrogenases were associated with QRL_C4A and QSL_D2A on chromosomes 4A and 2A, corresponding to root and shoot length traits under control and drought conditions without nano‑priming. These enzymes support lipid metabolism and energy production, which are essential for root and shoot growth under stress^101,102^. The genes encoding NADP‑dependent oxidoreductases were associated with QFW_D3A, which controls FW under drought conditions without nano‑priming. These enzymes participate in redox regulation mechanisms that help maintain biomass during drought stress^103,104^. The protease‑encoding genes associated with QFG %_D7B, linked to FG % under drought without nano‑priming, include enzymes such as prolyl endopeptidases. These proteases modulate peptide turnover and stress‑signaling pathways, processes known to influence germination under abiotic stress^105,106^. The lipase‑encoding genes linked to QFW_CN3B, which controls FW under control conditions with nano‑priming, include GDSL esterases/lipases. Although these enzymes have not previously been associated with wheat QTLs, they are known to participate in lipid remodeling and hormonal signaling, processes that contribute to improved seedling vigor^107–109^. The gene encoding O‑methyltransferases, associated with QRNo_DN1A on chromosome 1A, is linked to variation in root number under drought stress with nano‑priming. O‑methyltransferases are known to enhance antioxidant capacity and cell‑wall stability, thereby contributing to improved drought tolerance across plant species^110–112^. Finally, the gene encoded to Photosystem II stability associated with QFG %_DN7B related to FG % trait under drought treatment with nano priming. The Photosystem II stability factor was associated with improved germination under drought with nano-priming, consistent with evidence that PSII assembly and repair are critical for stress adaptation^113–116^. Collectively, these findings highlight diverse enzymatic pathways contributing to drought tolerance, seedling vigor, and metabolic resilience, and identify novel targets for functional validation in wheat breeding (Table S4 and S5).

Beyond enzymatic genes, several transcription‑factor families were also identified, highlighting their regulatory importance in drought adaptation. The candidate loci encode transcription factors such as C2H2‑type, AP2/ERF‑type, Agenet‑domain, BSD‑domain, and C3H1‑type proteins (Table S4 and S5). Such transcription factors may regulate key stress‑responsive pathways (e.g., ABA signaling, osmotic adjustment, and ROS scavenging), thereby modulating downstream genes involved in drought tolerance. The candidate gene annotated as a C2H2‑type zinc finger transcription factor was identified specifically under the control condition without nano‑priming and showed a significant association with QRL_C4A, which is linked to RL. Its detection under non‑stress conditions suggests that this transcription factor functions as a basal regulator of early root elongation. C2H2‑type zinc finger TFs are known to regulate root development and stress responses, consistent with their role in root elongation under control conditions^117–119^. Despite being detected only under optimal conditions, its association with RL_C suggests a basal architectural role rather than a stress‑induced function, consistent with the concept of pre‑stress priming. The candidate gene annotated as an AP2/ERF‑domain transcription factor was identified under drought conditions without nano‑priming and showed a significant association with QFW_D3A, linked to FW. This suggests a potential role in maintaining biomasses under limited water availability. Members of the AP2/ERF family are well known for their involvement in drought adaptation through the regulation of antioxidant capacity, osmotic adjustment, and growth maintenance. For example, overexpression of OsERF115 in rice improved water‑use efficiency, increased proline, and enhanced drought tolerance^120^. Likewise, MhERF113‑like in Malus improved drought survival and reduced oxidative damage, supporting the role of AP2/ERF TFs in stress mitigation^121^. These findings further confirm the regulatory role of AP2/ERF TFs in drought resilience. Overexpression of ERF109 enhances drought tolerance and biomass retention by modulating reactive‑oxygen‑species scavenging and auxin signaling^122^, whereas other AP2/ERF members such as ERF6, contribute to regulating growth and water loss under stress^123^. In wheat, TaERF‑3 has been shown to enhance drought resilience and maintain growth performance under water‑deficit conditions^124^. Similarly, ERF114 and ERF115 have been reported to regulate root‑growth plasticity and support adaptive development under environmental stress^125^. Collectively, the association of this gene with FW under drought is consistent with the established regulatory role of AP2/ERF transcription factors in maintaining biomass stability and coordinating stress‑responsive signaling during dehydration. This supports its potential function as a key regulator contributing to drought adaptation by sustaining water balance, antioxidant defense, and growth stability under adverse conditions. The gene encoding Agenet proteins, associated with QFW_D3B and QFW_DN3B, is linked to FW under both drought conditions. Agenet proteins are generally involved in chromatin remodeling and epigenetic regulation, suggesting a role in modulating stress‑responsive gene expression rather than direct enzymatic activity. Although direct evidence in wheat is limited, studies in model plants support this regulatory function. Chromatin modifications and remodeling regulate drought‑inducible genes and contribute to stress memory^126,127^. In Arabidopsis, dynamic histone changes activate stress‑responsive genes^128^, and Agenet/Tudor proteins act as histone‑mark readers that link chromatin state to transcriptional regulation^129^. The gene encoding BSD‑domain proteins, associated with QRNo_DN1A, is linked to RNo under drought condition with nano‑priming. BSD‑domain proteins are generally involved in transcriptional regulation, protein–protein interactions, and developmental control, rather than direct enzymatic activity. For example, in tomato, the BSD‑domain protein SlBSD1 functions as a transcription factor regulating vegetative growth and leaf senescence. Silencing SlBSD1 reduces plant growth and accelerates senescence, whereas its overexpression promotes growth and delays senescence^130^. Similarly, expression profiling of BSD‑domain genes in apricot across different developmental stages showed tissue‑specific and temporal expression patterns, indicating conserved roles in plant development and growth regulation^131^. Collectively, these findings indicate that BSD‑domain proteins can influence key developmental traits. Their association with RNo under drought conditions supports a potential role in modulating root architecture, thereby enhancing water acquisition and contributing to drought resilience in wheat. The gene encoding a C3H1‑type domain‑containing protein, associated with QCVt_DN2B, is linked to CVt under drought with nano‑priming. C3H1‑type proteins belong to the CCCH zinc‑finger family, which function as post‑transcriptional regulators involved in mRNA stability, processing, and stress‑responsive gene expression. Studies in plants demonstrate the role of CCCH‑type zinc‑finger proteins in abiotic stress tolerance and developmental regulation. For example, in Arabidopsis thaliana, the CCCH‑type protein AtTZF1 regulates mRNA decay and enhances drought and salt tolerance^132,133^. CCCH‑type zinc‑finger proteins, such as OsTZF1, enhance drought tolerance through post‑transcriptional regulation of stress‑related mRNAs^134^. Genome‑wide analysis in wheat further identified multiple ZFP genes with drought‑responsive expression, indicating a conserved functional role of the CCCH/ZFP family across cereals^135^. Collectively, these transcription factors coordinate major stress‑responsive pathways, including ABA signaling, osmotic adjustment, ROS scavenging, and epigenetic regulation, making them strong candidates for functional validation in wheat breeding (Table S4 and S5). Beyond enzymatic and transcriptional genes, candidates involved in signal transduction and post‑translational modification were also identified. Notably, the gene encoding a non‑specific serine/threonine protein kinase (STPK) was associated with QSL_DN1A, linked to SL under drought with nano‑priming. STPKs serve as central regulators of signal transduction by phosphorylating target proteins, thereby modulating stress responses and growth under environmental constraints. STPKs enhance drought tolerance by modulating ABA signaling, ROS scavenging, and growth regulation. In Arabidopsis thaliana, SnRK2‑type STPKs mediate ABA‑dependent responses, including stomatal closure and adjustments in shoot and root growth under water‑deficit conditions^136^. In rice, OsSAPK2 phosphorylates downstream transcription factors, enhancing antioxidant activity and maintaining shoot growth under drought conditions^137^. In wheat, serine/threonine protein kinases (STPKs), including members of the SnRK2 family, play pivotal roles in drought and salt‑stress signaling by regulating stress‑responsive gene expression and growth adaptation^138^. The association of this gene with SL suggests a regulatory role in shoot elongation under drought, potentially integrating nano‑priming‑induced signaling pathways to support growth under water‑limited conditions (Table S4 and S5). Overall, the functional annotation of the candidate genes highlights core enzymatic, regulatory, and signaling pathways underlying drought tolerance and early development in wheat, identifying key targets for future breeding and stress‑resilience strategies.

The exploration of expression profiles of the identified genes showed differential responses including three main transcriptional responses to drought: upregulation, downregulation, and stable expression of genes (Table S6, Fig 5). Upregulated genes enhance osmotic adjustment, antioxidant defense, and stress signaling, consistent with previous reports on drought-responsive genes such as DREB, LEA, and HSP^139,140^. Downregulated genes indicate a strategic shift of resources from growth and metabolism toward essential stress adaptation^139^. Meanwhile, stably expressed genes likely provide a constitutive defense mechanism, maintaining physiological stability under water deficit^140^. Collectively, these patterns reflect a coordinated transcriptional strategy that allows wheat seedlings to efficiently cope with drought stress.

## Conclusions

This study successfully identified several quantitative trait loci (QTLs) associated with drought tolerance during germination and early seedling stages in wheat under both unprimed and nano-priming conditions. The detected loci on chromosomes 4A, 3A, 3B, 2A, 7B, 1A, and 2B highlighted key genomic regions involved in germination traits, root architecture, shoot development, and overall seedling vigor under water-deficit stress. Notably, QTLs located on chromosome 3B exhibited pleiotropic effects, suggesting their broad regulatory roles in early-stage stress adaptation. The identification of drought-related QTLs under nano-priming conditions indicates that ZnO-NPs priming can enhance the activation of stress-responsive genes, resulting in improved germination and seedling establishment under drought. These results demonstrate that ZnO-NPs effectively improves wheat’s drought tolerance, which is crucial for enhancing one of the most important grain crops worldwide and addressing global food shortages. Collectively, integrating ZnO-NPs priming with conventional breeding approaches could accelerate the development of drought-tolerant wheat cultivars. While genetic variation and heritability estimates highlight the potential for selection, nano-priming provides an additional physiological advantage by improving germination and early vigor under water deficit. Thus, combining genetic improvement with seed nano-priming strategies offers a sustainable and complementary pathway to enhance wheat performance in drought-prone environments. However, given the relatively small population, the identified loci should be considered as candidate regions and require further validation in larger and independent populations before their application in breeding programs.

## Supplementary Information

Supplementary Information 1.

Supplementary Information 2.

## Data Availability

“The datasets generated during and/or analyzed during the current study are available from the corresponding author on reasonable request”.
