# Supplementary material for: Quantitative trait loci associated with drought stress tolerance in wheat primed with zinc oxide nanoparticles at seed germination and seedling stages
Source: Sci Rep. 2026 Apr 6;16:11612. doi: 10.1038/s41598-026-43113-7 (PMC13057053; doi:10.1038/s41598-026-43113-7)
Supplement: Supplementary file 1 — Supplementary Information 1. [file 41598_2026_43113_MOESM1_ESM.pptx]

## Slide 1
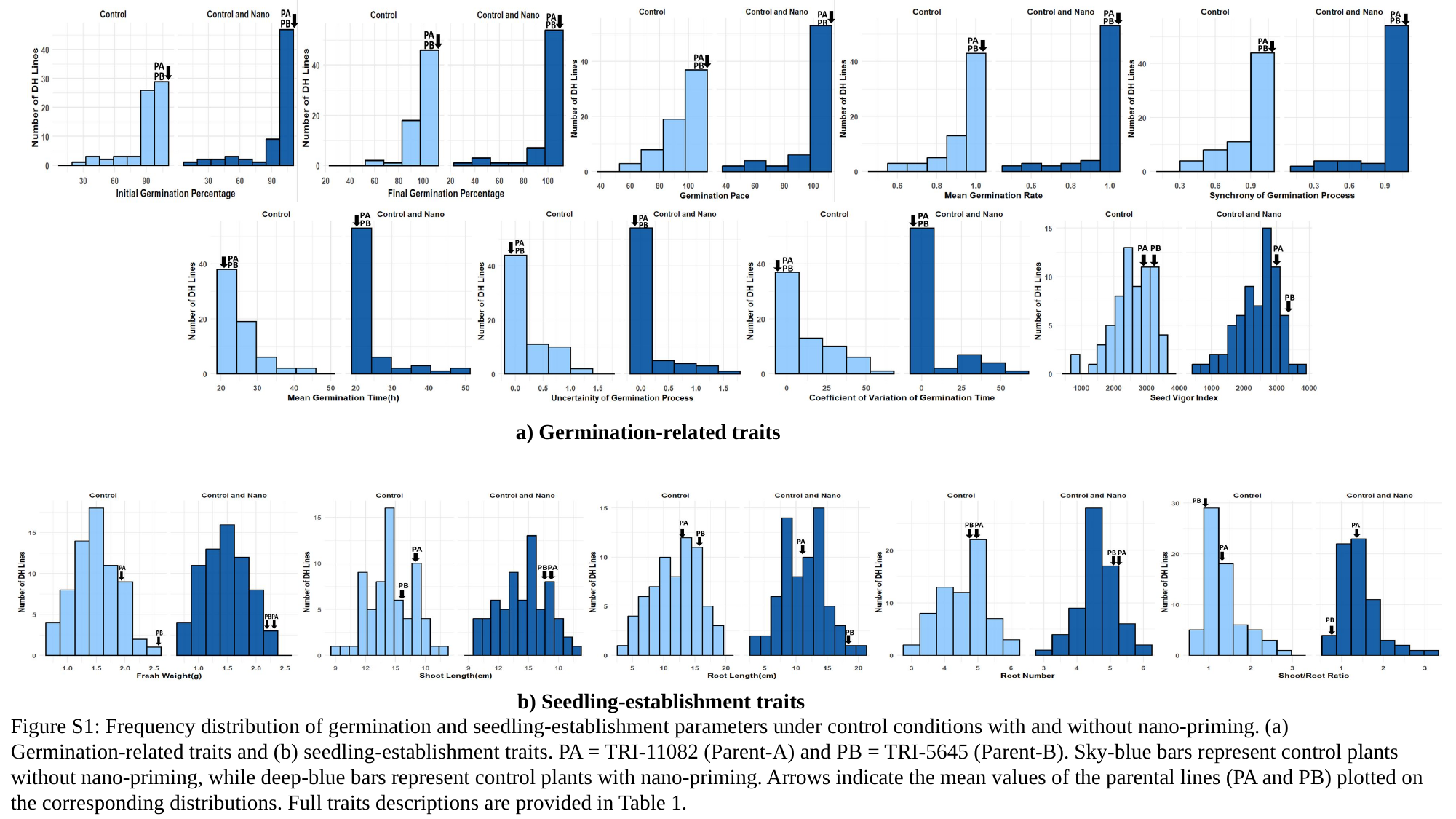

a) Germination-related traits
b) Seedling-establishment traits
Figure S1: Frequency distribution of germination and seedling‑establishment parameters under control conditions with and without nano‑priming. (a) Germination‑related traits and (b) seedling‑establishment traits. PA = TRI‑11082 (Parent‑A) and PB = TRI‑5645 (Parent‑B). Sky‑blue bars represent control plants without nano‑priming, while deep‑blue bars represent control plants with nano‑priming. Arrows indicate the mean values of the parental lines (PA and PB) plotted on the corresponding distributions. Full traits descriptions are provided in Table 1.

## Slide 2
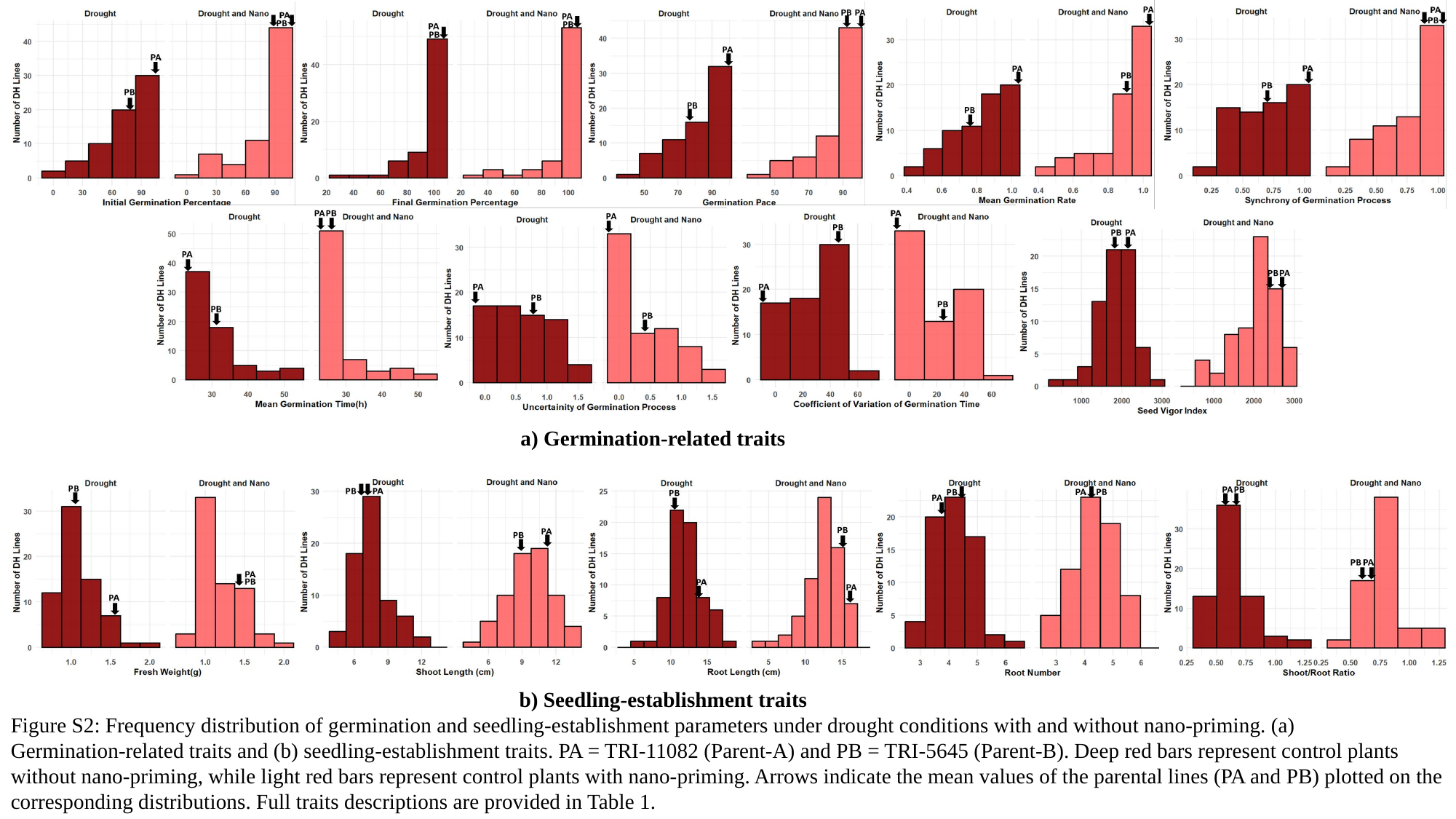

a) Germination-related traits
b) Seedling-establishment traits
Figure S2: Frequency distribution of germination and seedling‑establishment parameters under drought conditions with and without nano‑priming. (a) Germination‑related traits and (b) seedling‑establishment traits. PA = TRI‑11082 (Parent‑A) and PB = TRI‑5645 (Parent‑B). Deep red bars represent control plants without nano‑priming, while light red bars represent control plants with nano‑priming. Arrows indicate the mean values of the parental lines (PA and PB) plotted on the corresponding distributions. Full traits descriptions are provided in Table 1.

## Slide 3
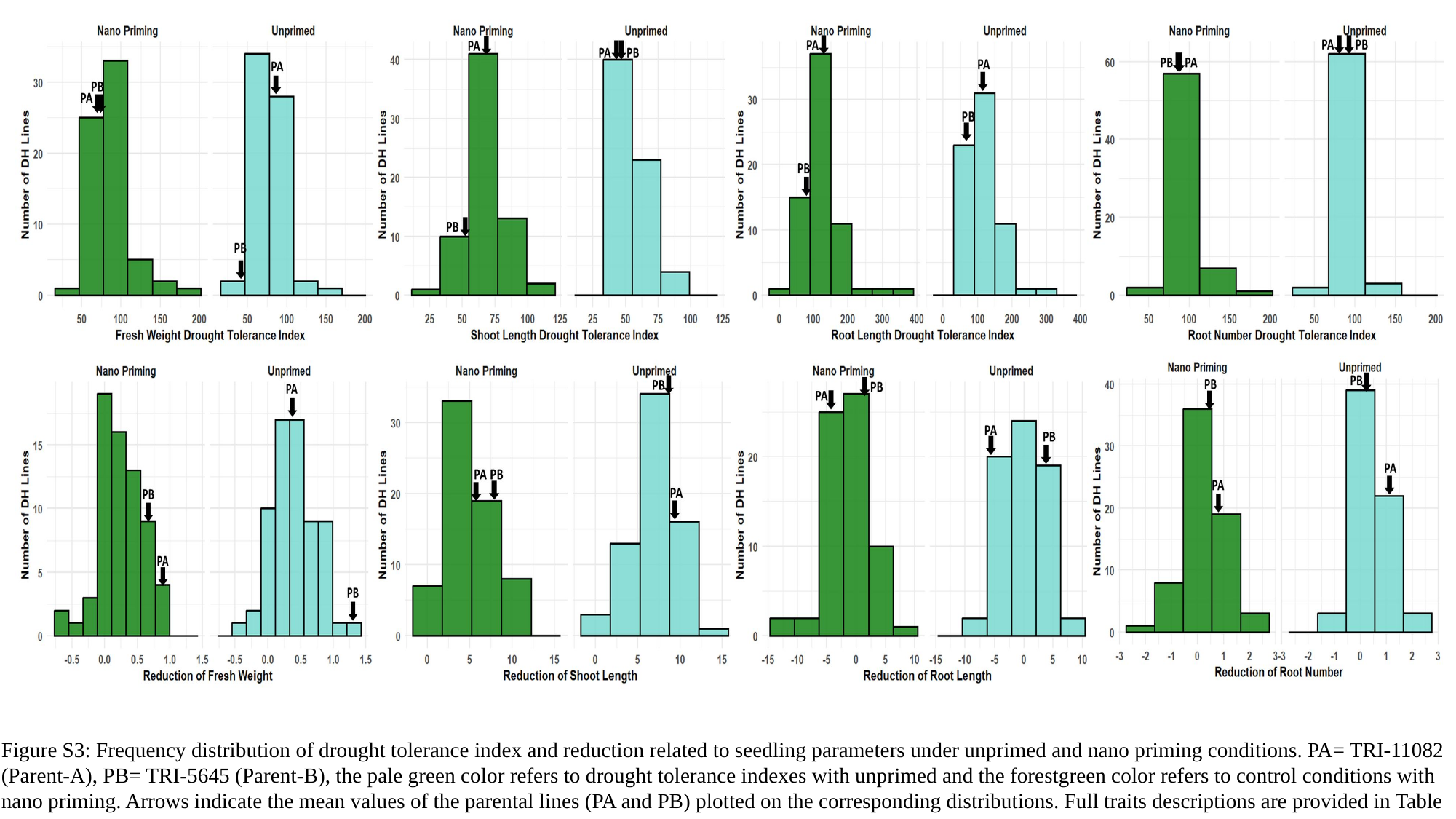

Figure S3: Frequency distribution of drought tolerance index and reduction related to seedling parameters under unprimed and nano priming conditions. PA= TRI-11082 (Parent-A), PB= TRI-5645 (Parent-B), the pale green color refers to drought tolerance indexes with unprimed and the forestgreen color refers to control conditions with nano priming. Arrows indicate the mean values of the parental lines (PA and PB) plotted on the corresponding distributions. Full traits descriptions are provided in Table 1.

## Slide 4
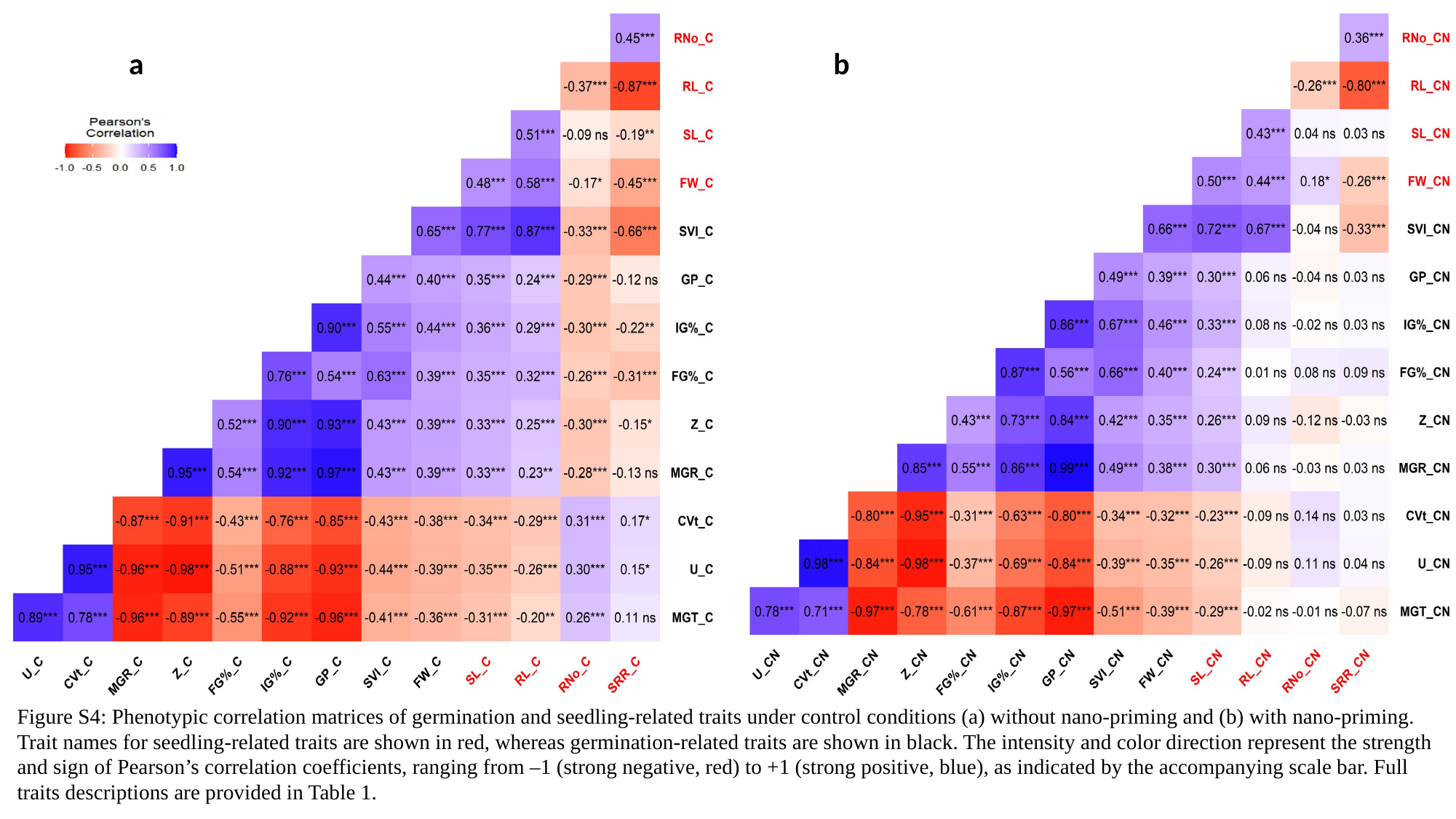

b
a
Figure S4: Phenotypic correlation matrices of germination and seedling‑related traits under control conditions (a) without nano‑priming and (b) with nano‑priming. Trait names for seedling‑related traits are shown in red, whereas germination‑related traits are shown in black. The intensity and color direction represent the strength and sign of Pearson’s correlation coefficients, ranging from –1 (strong negative, red) to +1 (strong positive, blue), as indicated by the accompanying scale bar. Full traits descriptions are provided in Table 1.

## Slide 5
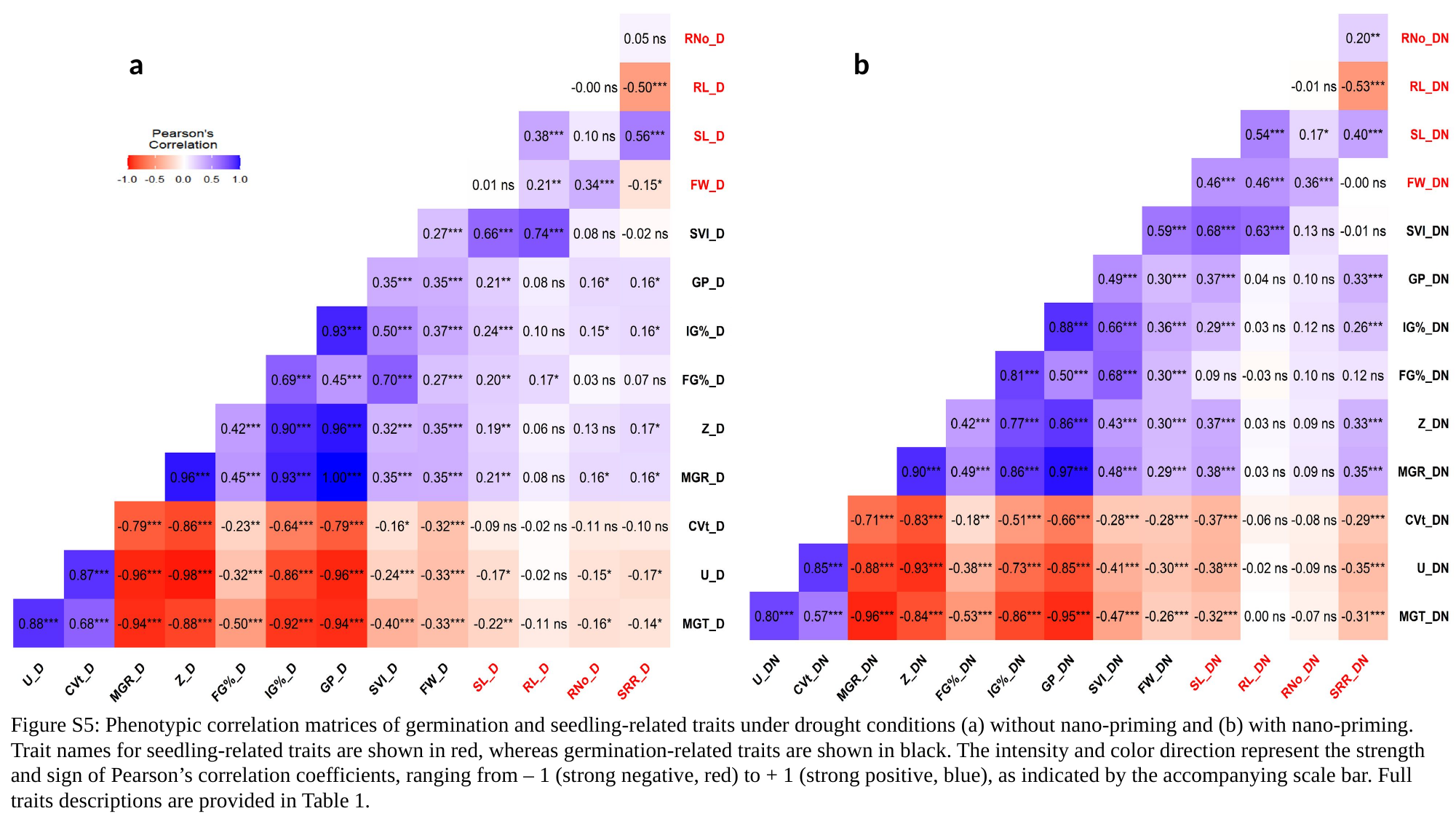

a
b
b
Figure S5: Phenotypic correlation matrices of germination and seedling‑related traits under drought conditions (a) without nano‑priming and (b) with nano‑priming. Trait names for seedling‑related traits are shown in red, whereas germination‑related traits are shown in black. The intensity and color direction represent the strength and sign of Pearson’s correlation coefficients, ranging from – 1 (strong negative, red) to + 1 (strong positive, blue), as indicated by the accompanying scale bar. Full traits descriptions are provided in Table 1.

## Slide 6
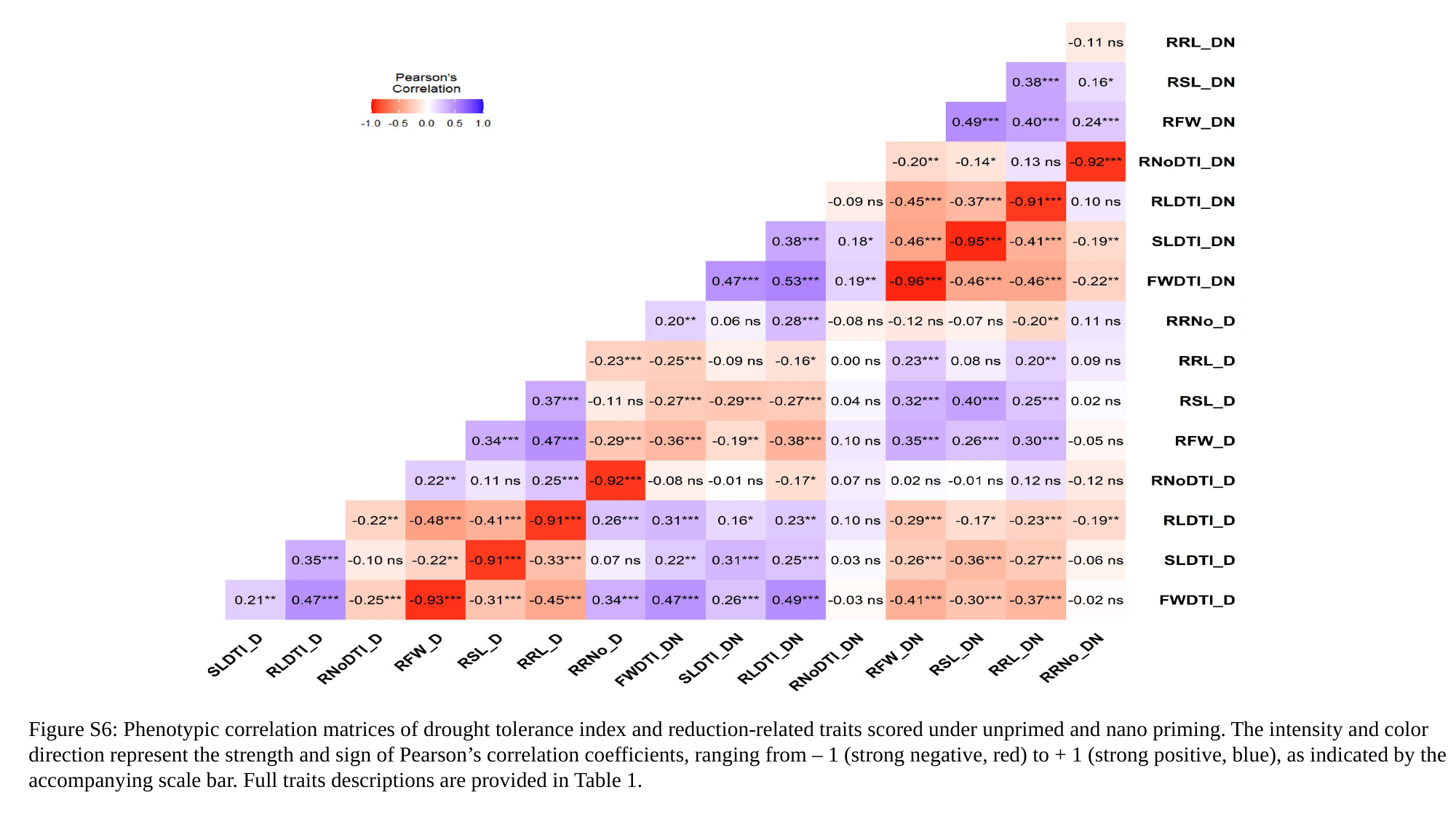

Figure S6: Phenotypic correlation matrices of drought tolerance index and reduction-related traits scored under unprimed and nano priming. The intensity and color direction represent the strength and sign of Pearson’s correlation coefficients, ranging from – 1 (strong negative, red) to + 1 (strong positive, blue), as indicated by the accompanying scale bar. Full traits descriptions are provided in Table 1.

## Slide 7
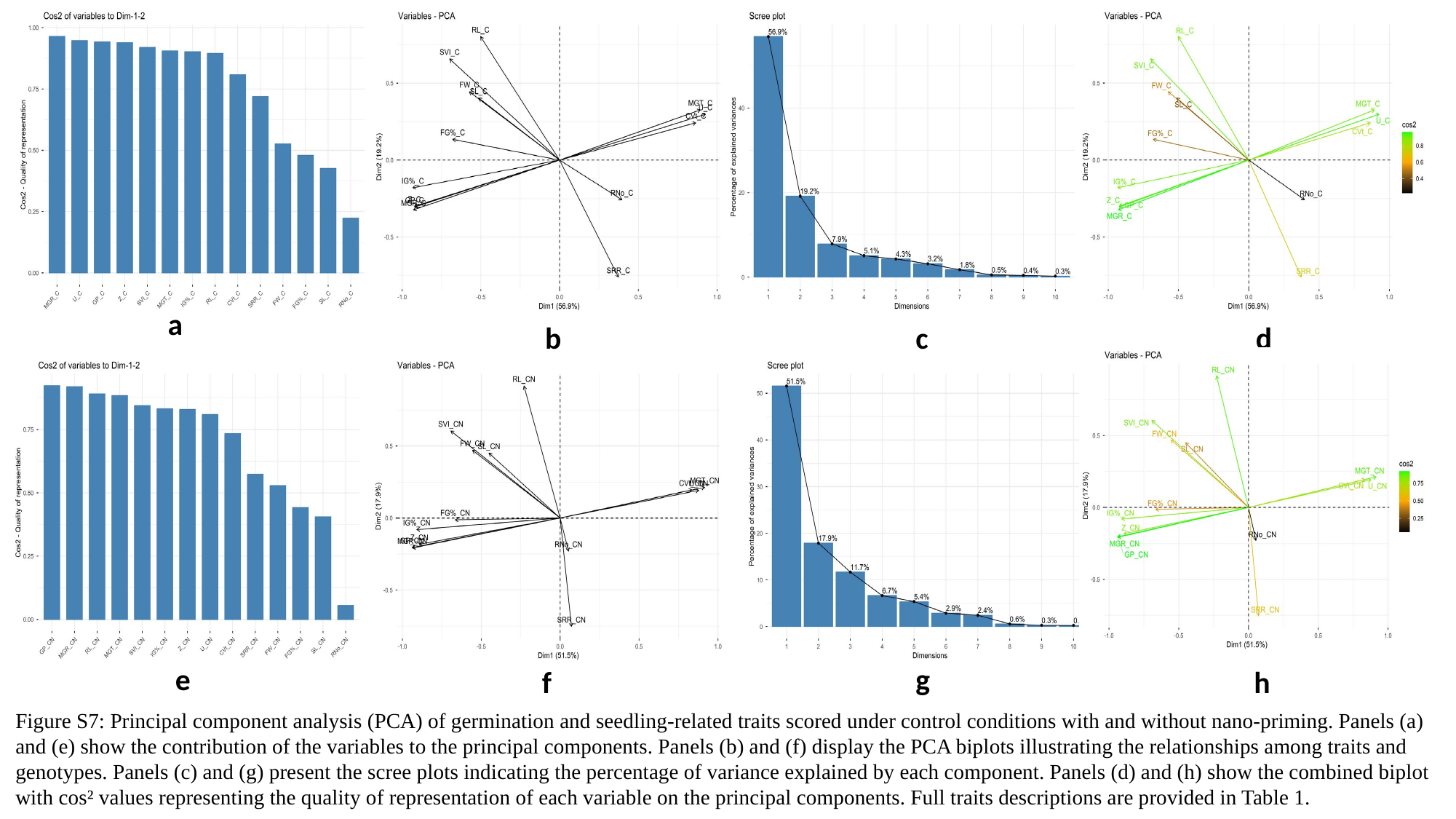

a
b
c
d
e
g
f
h
Figure S7: Principal component analysis (PCA) of germination and seedling‑related traits scored under control conditions with and without nano‑priming. Panels (a) and (e) show the contribution of the variables to the principal components. Panels (b) and (f) display the PCA biplots illustrating the relationships among traits and genotypes. Panels (c) and (g) present the scree plots indicating the percentage of variance explained by each component. Panels (d) and (h) show the combined biplot with cos² values representing the quality of representation of each variable on the principal components. Full traits descriptions are provided in Table 1.

## Slide 8
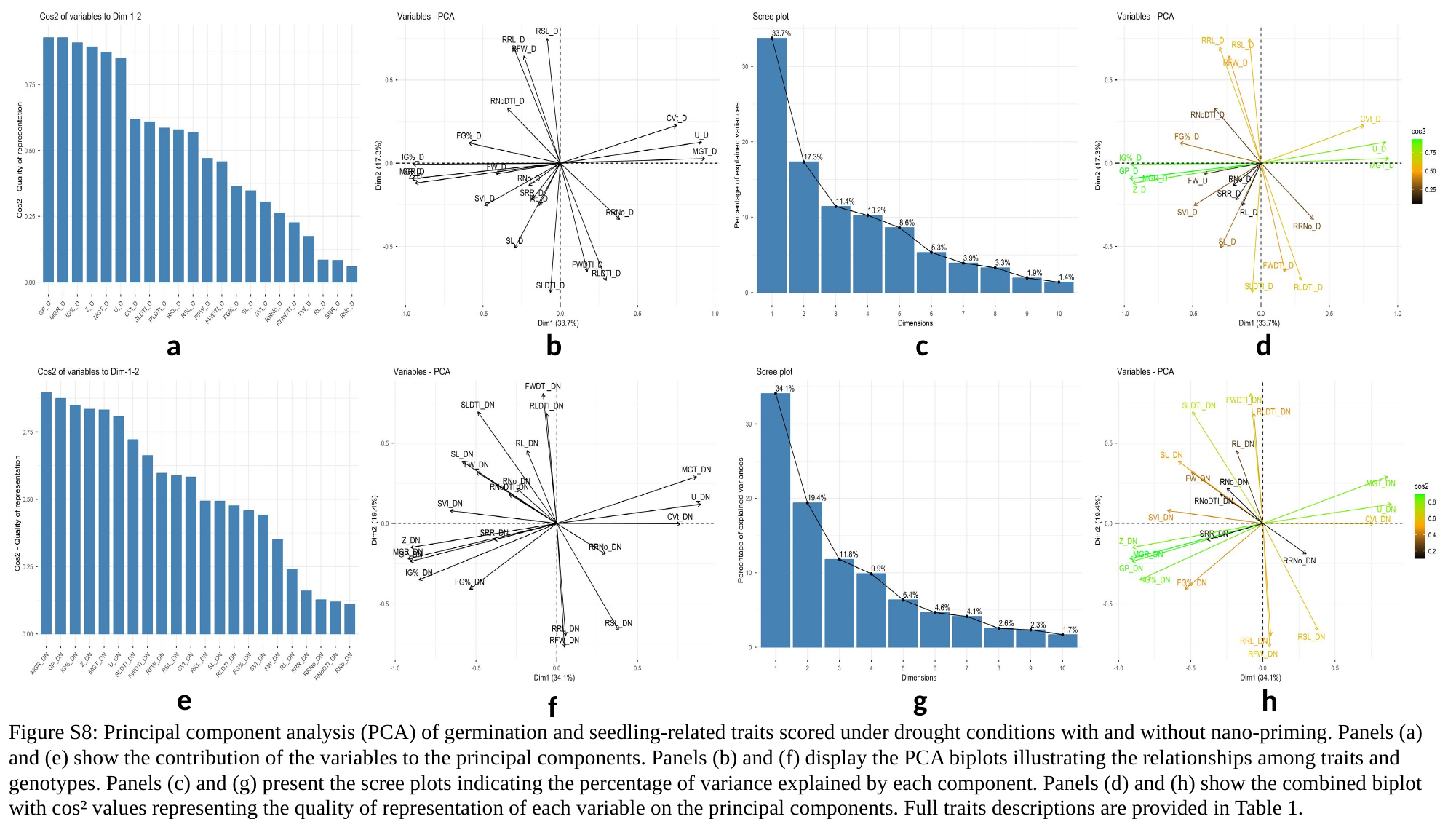

a
b
c
d
e
h
g
f
Figure S8: Principal component analysis (PCA) of germination and seedling‑related traits scored under drought conditions with and without nano‑priming. Panels (a) and (e) show the contribution of the variables to the principal components. Panels (b) and (f) display the PCA biplots illustrating the relationships among traits and genotypes. Panels (c) and (g) present the scree plots indicating the percentage of variance explained by each component. Panels (d) and (h) show the combined biplot with cos² values representing the quality of representation of each variable on the principal components. Full traits descriptions are provided in Table 1.
